# Considering a Utility-Centric Framework Based on “Minimum Orthophosphate” Criteria for Mitigation of Elevated Cuprosolvency in Drinking Water

**DOI:** 10.1021/acs.est.4c00583

**Published:** 2024-03-12

**Authors:** Rebecca B. Kriss, Emily Smith, Grace Byrd, Michael Schock, Marc A. Edwards

**Affiliations:** # Virginia Polytechnic and State University, Civil and Environmental Engineering, 418 Durham Hall, Blacksburg, Virginia 24061, United States; ‡ Retired, Cincinnati, Ohio 45230, United States

**Keywords:** Drinking water, copper corrosion, cuprosolvency, orthophosphate, corrosion control treatment

## Abstract

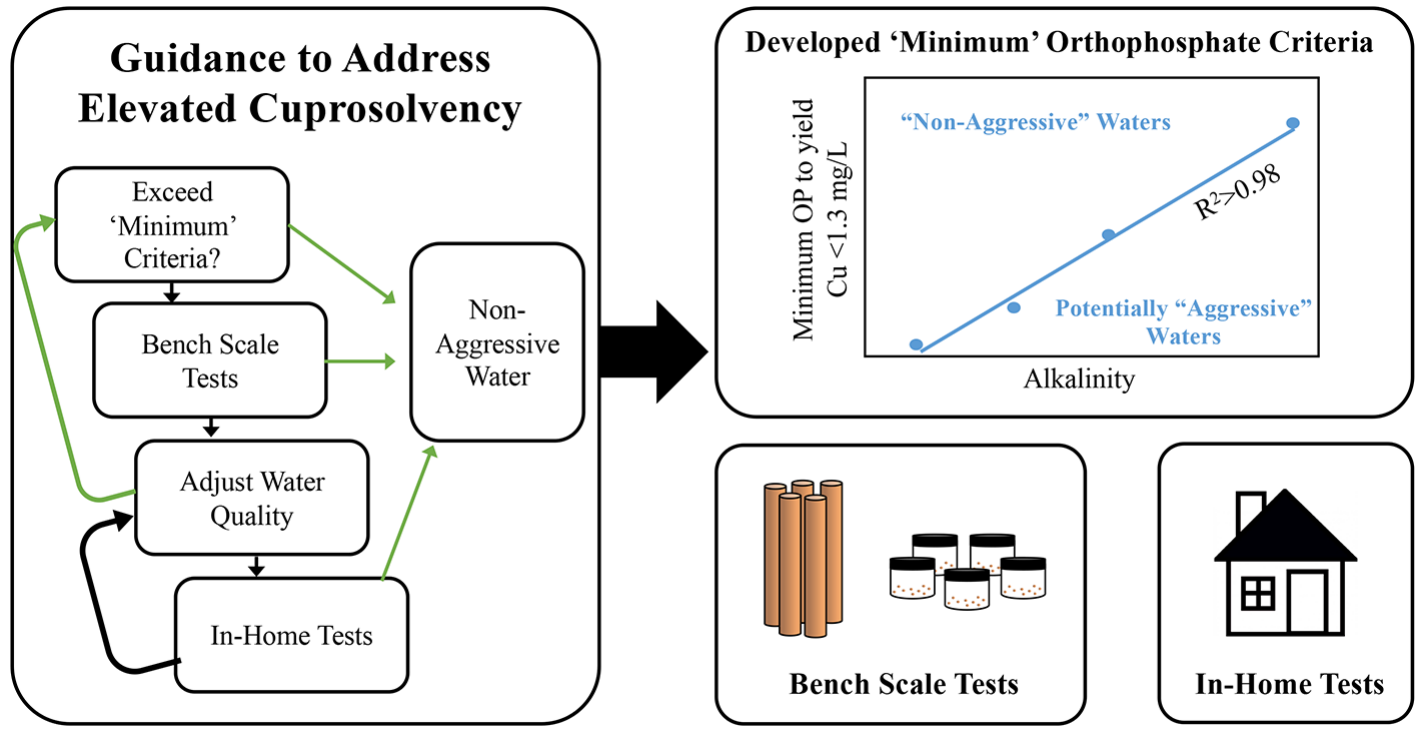

Gaps in the United States Environmental Protection Agency (US EPA) Lead and Copper Rule (LCR) leave some consumers and their pets vulnerable to high cuprosolvency in drinking water. This study seeks to help proactive utilities who wish to mitigate cuprosolvency problems through the addition of orthophosphate corrosion inhibitors. The minimum doses of orthophosphate necessary to achieve acceptable cuprosolvency in relatively new copper pipe were estimated as a function of alkalinity via linear regressions for the 90th, 95th, and 100th percentile copper tube segments (*R*^2^ > 0.98, *n* = 4). Orthophosphate was very effective at reducing cuprosolvency in the short term but, in some cases, resulted in higher long-term copper concentrations than the corresponding condition without orthophosphate. Alternatives to predicting “long-term” results for copper tubes using simpler bench tests starting with fresh Cu(OH)_2_ solids showed promise but would require further vetting to overcome limitations such as maintaining water chemistry and orthophosphate residuals and to ensure comparability to results using copper tube.

## Abstract 2

Gaps in the LCR may leave residents exposed to copper in drinking water. This work develops guidance to address cuprosolvency based on “minimum” orthophosphate criteria and considers benefits and drawbacks of various cuprosolvency tests and orthophosphate use.

## 1 Introduction

Cuprosolvency is the tendency of waters to cause release of soluble copper from pipes to water.^1^ Elevated copper can cause aesthetic concerns (e.g., fixture staining) and both acute and long-term health concerns for consumers and their pets.^2−4^ Cuprosolvency is one manifestation of copper corrosion, which can also include pinhole leaks (i.e., pitting), wall thinning, and erosion corrosion, all of which have unique physicochemical etiologies.^5^

To reduce problems with elevated cuprosolvency throughout community water systems, the 1991 United States Environmental Protection Agency (EPA) Lead and Copper Rule (LCR) set an action level (AL) of 1.3 mg/L for the 90th percentile copper concentration.^3,6−8^ However, the LCR only applies to municipal water systems and focuses sampling efforts on older homes (pre-1986) at greatest risk for elevated lead—not on the homes with newer plumbing that is more likely to have elevated cuprosolvency.^9−11^

In the three decades since the LCR was implemented, the minimum age of the copper pipes in the pre-1986 sampling pool has increased from about 5 to 35 years, further exacerbating the disconnect between metal release from new copper and compliance monitoring results. Consequently, some utilities can have serious problems with metal release from new copper without triggering any requirement for corrosion control based on LCR monitoring in older homes. Moreover, residents with private wells and other nonresidential buildings are not regulated under the LCR.^1,10,12,13^ These gaps can leave private well owners and buildings with new plumbing in need of remedies for persistent high cuprosolvency.^2,9,14^

The 2015 National Drinking Water Advisory Council (NDWAC) consensus statement proposed updates to the LCR to address these deficiencies. The NDWAC asserted that many potable waters are inherently nonaggressive to new copper and utilities with such water should be exempted from burdensome LCR sampling.^15^ To classify waters as “non-aggressive,” water quality parameters meeting standards for low cuprosolvency should be defined for new plumbing.

It is generally thought that, as new copper pipes age in contact with drinking water, relatively soluble scales such as cupric hydroxide [Cu(OH)_2_] initially form and control the maximum cuprosolvency in water.^1^ Upon aging, these cupric hydroxide solids are thought to transition to less soluble solids (e.g., tenorite [CuO], malachite [Cu_2_(OH)_2_CO_3_], and cupric phosphates).^1,6,9−11,16−19^ Many water quality parameters such as oxidants, pH, chloride, sulfate, hardness, temperature, natural organic matter (NOM), microorganisms, and nitrogen, sulfur, iron species, etc. may affect the rate of scale transitions, and therefore cuprosolvency, through complex reactions including redox, complexation, crystallization, and crystal poisoning.^1,16,20−27^ It is believed that transitions from more soluble to less soluble solids can occur in timespans of minutes to decades, depending on water chemistry, and are irreversible in normal situations with relatively stable water quality.^9^

The NDWAC proposed demarcating “non-aggressive” waters based on two standard corrosion control approaches at water utilities: dosing of orthophosphate inhibitors or adjusting pH/alkalinity.^15,28^ This approach was designed to be conservative, the goal being that very few homes in waters labeled “non-aggressive’ would test above the 1.3 mg/L copper AL after a few months of exposure to the water. Ultimately, the most recent LCR revisions did not follow these NDWAC recommendations attempting to address cuprosolvency concerns. The framework can still provide guidance to building managers, residents, or utilities who wish to address these concerns on a voluntary basis.^29,30^

This study aims to guide voluntary action addressing cuprosolvency concerns in new copper plumbing by identifying the minimum orthophosphate doses needed to maintain copper below 1.3 mg/L as a function of the water’s alkalinity.^1,9,31,32^ This proposed NDWAC approach was vetted and refined using experiments with copper pipe. Simpler and less expensive laboratory cuprosolvency tests monitoring the aging of fresh cupric solids were studied as part of this evaluation.

## 2 Methods

Bench scale cuprosolvency testing using copper tube segments was used to establish minimum doses of orthophosphate to render a water “non-aggressive” to copper as a function of pH and alkalinity. “Non-aggressive” is defined as a water which would reduce soluble copper below the 1.3 mg/L AL after 5.5 months or less of exposure for a typical copper pipe.

### 2.1 Copper Tube Cuprosolvency Testing

Copper tube cuprosolvency tests were similar to that of Kriss and Edwards and utilized 8.5” segments of new 1/2” diameter type M copper tubing.^24^ Each test utilized 5 pipe segments purchased from 4 manufacturers (20 segments total). Pipes were stoppered at one end, filled with water, and covered during storage to prevent air exchange. Water changes were performed using a dump and fill protocol three times per week to simulate flow inside pipes with periods of longer stagnation due to COVID-19 related laboratory shutdowns. Experiments were carried out using synthetic drinking waters under conditions known to cause high cuprosolvency, including cooler temperature (15 °C) and the presence of trace NOM (0.2 mg/L as fulvic acid from International Humic Substance Society) and high sulfate (200 mg/L).^6,9,16,33,34^ Even though this represents a relatively low total organic carbon (TOC), the NOM concentration was near or above those reported to have substantial effects on cuprosolvency, and fulvic acid is known to be more active than particulate or colloidal NOM which may be found in drinking water.^9,24,27,33,34^ Experiments utilized four orthophosphate doses in each of four waters with combinations of pH and alkalinity based on the intermediate value used in Kriss and Edwards (2023) that roughly approximated the boundaries of the NDWAC pH bin criteria (16 total waters, Table 1).^15,24^ A total of 320 tube segments (16 waters × 20 segments) were tested. Periodic composite (combined water from all tubes for a water condition) and full (individual tubes) samplings were performed, and samples were analyzed for total copper concentrations released into the water.

**Table 1:** Summary of Synthetic Water Conditions for Cuprosolvency Testing in Copper Tube

Alkalinity (mg/L as CaCO_3_)	Dissolved Inorganic Carbon (mg C/L)^b^	pH	Orthophosphate Dose (mg/L as P)
35	4.2	7.25	0
			0.06
			0.13
			0.26^a^
100	12	7.25	0
			0.2
			0.4
			0.6
250	30	7.5	0
			0.5
			1
			1.5
500	60	8	0
			1.5
			2
			2.5

a After week 20, the orthophosphate dose was decreased to 0.03 mg/L as P.

b The approximate dissolved inorganic carbon concentration (DIC) was calculated based on the pH and alkalinity of the test water.

### 2.2 Cuprosolvency Testing Using Fresh Copper Solids

An attempt was made to determine the minimum orthophosphate dose using freshly prepared cupric solids. A simplified cuprosolvency test was used based on Edwards et al. (2001),^6^ but without the automatic titration method of prior testing,^35,36^ to facilitate easier application by utilities and other stakeholders. Initial experiments utilized analogous water chemistries as in copper tube experiments (Table 1) with additional NOM (0.4 mg/L) to reflect that the NOM threshold for increasing cuprosolvency observed in a companion study was between 0.2 and 0.5 mg/L NOM as fulvic acid.^24^ Background water chemistry components and orthophosphate were added prior to the cupric nitrate addition. Cupric solid formation was initiated with the addition of 0.5 mM cupric nitrate with stirring at 200 rpm. The pH was adjusted to the target (± 0.05) within 5 min and maintained within ±0.25 of the target thereafter using 1 M NaOH or nitric acid. Aliquots were stored in 125 mL plastic bottles until sampling at predetermined intervals (*n* = 1 for samples up to 1 week; n = 5 replicates for 2-week and 1-month samplings). Bottles were shaken at each sampling time. Dissolved copper was determined by filtering samples through 0.45 μm nylon syringe filters (Whatman) prior to analysis. Composite solid samples were collected after 2 weeks and 1 month of aging using a vacuum filter (Whatman 0.45 μm).

Additional tests examined orthophosphate dosing strategies. Experiments utilized 1 L of test water at pH 7.5 with 250 mg/L as CaCO_3_ alkalinity, 0.2 mg/L NOM, and 200 mg/L sulfate. A larger volume was utilized for this test to facilitate residual orthophosphate testing and dosing. First, a 1.5 mg/L as P orthophosphate residual was maintained by adding an initial dose of orthophosphate with additions at each sampling time to replenish orthophosphate to the target level. The second experiment employed one initial orthophosphate addition of 4.3 mg/L as P, equivalent to the total orthophosphate added in the previous test. Samples were filtered using a 0.45 μm pore size filter for dissolved copper analysis.

### 2.3 Analytical Methods

Alkalinity and pH were determined via standard methods.^37^ Hach methods 8506 and 8048 were used to track copper and orthophosphate concentrations (Hach DR3900). All samples used for statistical analysis were analyzed using Atomic Absorption Spectroscopy (AAS, PerkinElmer 5100 PC AAS) via method 3111 B (copper) and ICP-MS/MS (Thermo Scientific iCAP RQ ICP-MS) via method 3125 B (copper, phosphorus, and other metals), after acidification to 2% nitric acid by volume for at least 16 h.^37^ QA/QC was performed for AAS and ICP-MS/MS after every 10 samples via the measurement of standards. All materials were of reagent grade quality.

### 2.4 Data Analysis

Statistical analyses were performed on full sampling results from individual tube segments using R statistical software (version 4.2.1) and the ANOVA function with a significance threshold of *p* ≤ 0.05. Interpolation of full sampling results (described in section S1, Figures S1–S6) facilitated development of linear regressions identifying minimum orthophosphate doses required to yield copper below the 1.3 mg/L AL for given alkalinities considering the highest, second highest, and third highest copper concentrations (herein called the 90th, 95th, and 100th percentile, for the 20 replicates tested). Copper solubility at thermodynamic equilibrium was predicted using MINEQL+ (version 4.6) following the method in section S2.

## 3 Results and Discussion

### 3.1 Proposed Utility Framework for Cuprosolvency Mitigation

**Figure 1:**
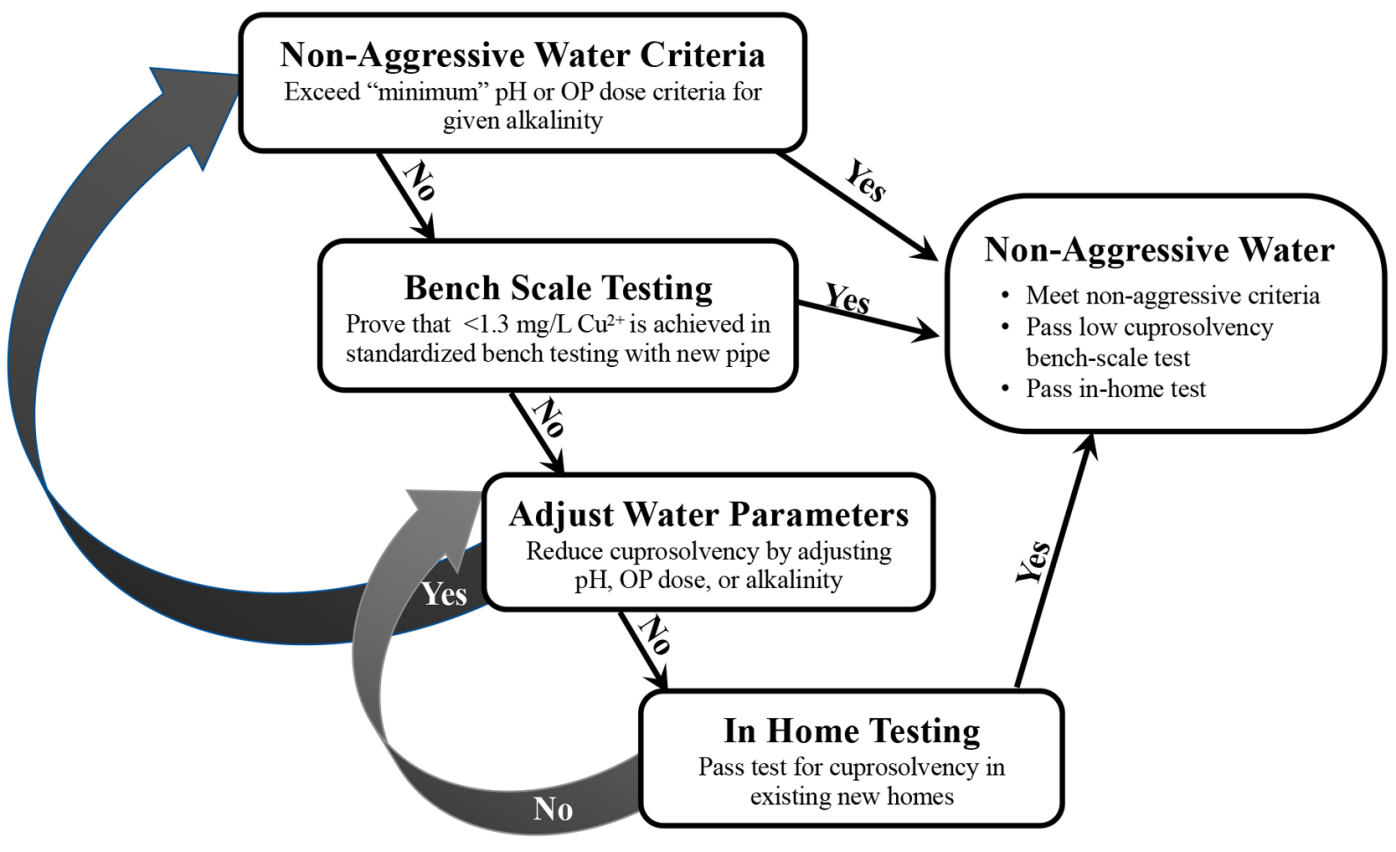
A proposed decision-making framework for utilities to aid with determination of “non-aggressive” waters for new copper piping.

A framework was developed, stemming from NDWAC consensus statement suggestions, to guide proactive utilities and other stakeholders in addressing cuprosolvency issues (Figure 1). The NDWAC proposed that minimum orthophosphate doses for given alkalinities could yield “non-aggressive” waters and almost always keep copper below the 1.3 mg/L AL even in relatively new homes. They put forth example values but noted the need for further verification.^15^ Such “minimum” doses could serve as criteria for effective cuprosolvency control without requiring burdensome sampling to verify performance. We refined that “minimum” orthophosphate dose criteria (section 3.1.3 and Figure 3) and used it as the basis for our framework.

We posited that if a utility did not meet the “minimum” criteria, which was designed to have a safety factor, they could prove that their waters were nonaggressive using verification testing. Such testing could be done for their particular water using simple bench-scale tests, such as those presented in section 2.1, with worst case new copper pipe or through traditional field sampling, as prescribed under the LCR, but sampling in homes with new copper pipe. In the absence of regulations these approaches might someday qualify as best practices identified in “Recommended Standards for Water Works” or similar programs.^38^

#### 3.1.1 Effects of Orthophosphate in Copper Tube Cuprosolvency Testing

**Figure 2:**
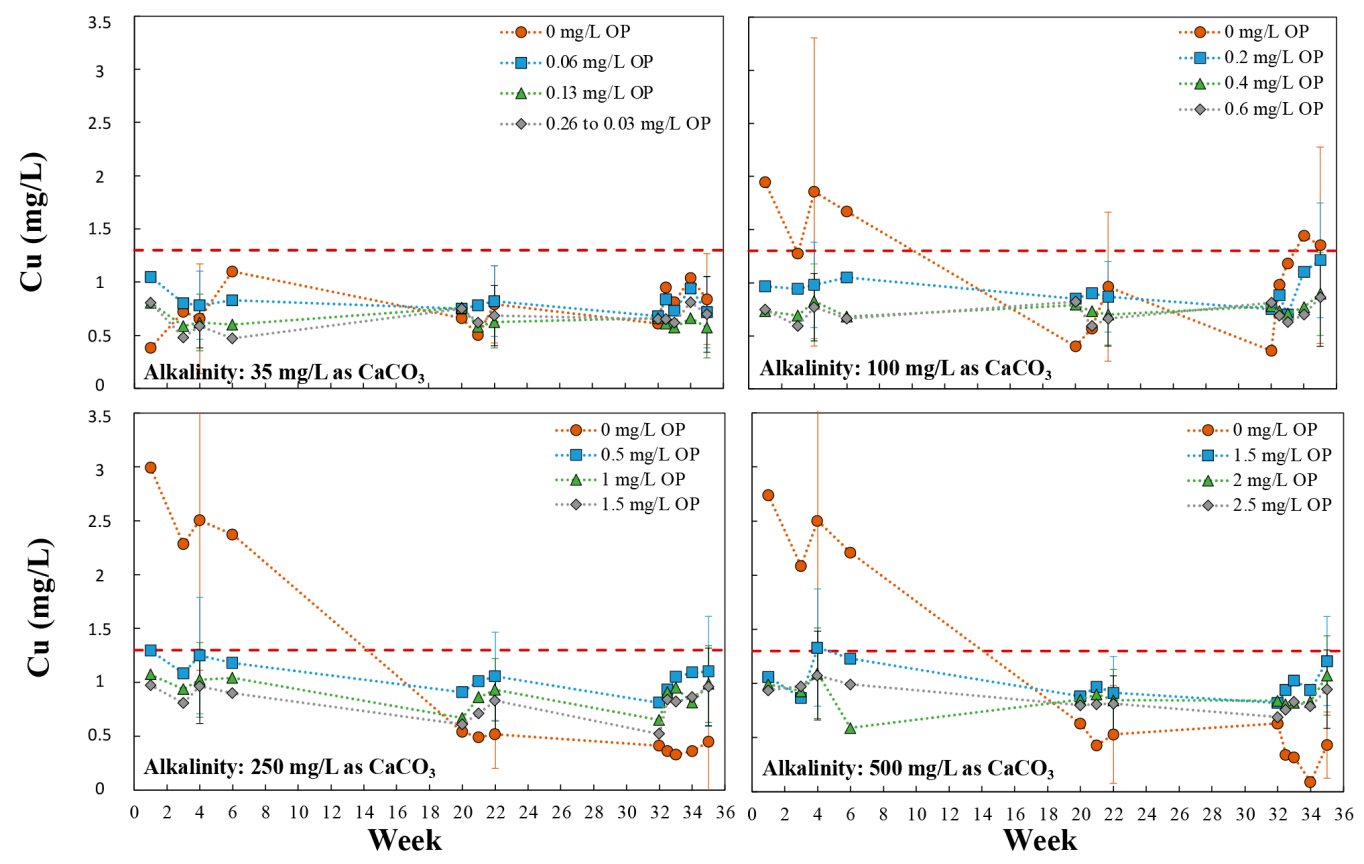
Mean copper release from new copper pipes treated with varying levels of orthophosphate corrosion control (*n* = 20 for weeks 4, 22, and 35; *n* = 1 composite for all other times). Error bars represent one standard deviation. Red dotted lines denote the 1.3 mg/L action level. All orthophosphate (OP) doses are in mg/L as P.

Orthophosphate addition to the synthesized water was successful in yielding average copper concentrations below the 1.3 mg/L AL for all waters after the end of week 1, except in one condition, which was only 2.4% above the AL (Figure 2). Consistent with the underlying NDWAC “minimum orthophosphate” criteria hypothesis, higher orthophosphate doses generally resulted in lower cuprosolvency. The exception was the lowest alkalinity, where elevated copper was not a problem, so no orthophosphate would be required.

Most orthophosphate treated waters yielded average copper below those without orthophosphate within the first 6 weeks of treatment, but as expected, in some waters orthophosphate yielded higher long-term cuprosolvency. For example, initial copper concentrations (first 6 weeks) at pH 7.5 and 250 mg/L as CaCO_3_ alkalinity with orthophosphate were less than half that without orthophosphate (0.94–1.07 mg/L vs 2.29–2.99 mg/L), but after 22 weeks the condition with orthophosphate had higher copper than without orthophosphate (0.93 vs 0.52 mg/L). Such long -term detriments of orthophosphate dosing have been noted in prior literature and are attributed to rapid formation of low solubility cupric phosphates, but inhibited formation of very low solubility minerals like tenorite or malachite that may form over a period of months, years, or decades.^1,6,7,9−11,16−19,31,32,39,40^ Because of potential higher long-term copper release and concerns about expense and sustainability of phosphate use, utilities could instead choose to prove the efficacy of pH/alkalinity corrosion control criteria in their water.^9^ Even so, the speed and reliability of orthophosphate corrosion control may be beneficial in many cases, such as those in a companion study where elevated cuprosolvency persisted in laboratory studies (>AL) for 8.75–19.75 months for some water conditions, and in 5 of 361 utilities surveyed (560–1,270 ppb) over a period of at least three decades in homes built before 1986.^24^

Visual observations of scales were consistent with these results, with blue-green scales characteristic of malachite present in high alkalinity pipes without orthophosphate whereas no blue-green scales were observed in pipes conditioned with orthophosphate.^9^

#### 3.1.2 Manufacturer Differences in Copper Tube Cuprosolvency Testing

Contrary to expectations that most off-the-shelf copper tubes would yield consistent and relatively similar cuprosolvency, substantial variability was observed among tubes of different manufacturers (Figures S9–S12). Mean copper concentrations from one manufacturer were 0.39 to 1.26 mg/L (average 69%, range 51–88%) lower than those of the other three manufacturers and were significantly different (*p* < 0.05, *n* = 5 per manufacturer) for all OP conditions and times tested. In contrast, mean copper concentrations comparing the other manufacturers for these conditions were only significantly different for 17.6% of cases, with an average difference of 10% (range 0–46%). These cuprosolvency differences are consistent with significant manufacturer differences observed in a companion study and may result from manufacturing and atmospheric differences creating different oxide films initially present on the pipes.^24,41^ Using tube from the outlier manufacturer in cuprosolvency testing could be misleading, since it yielded significantly lower copper (<AL) for 19.4% of cases where the mean copper from other manufacturers exceeded the AL. This complication did not affect determination of “minimum” orthophosphate criteria using the 20 tubes from all four manufacturers presented in the next section.

#### 3.1.3 Determination of Minimum Orthophosphate Dose Criteria

**Figure 3:**
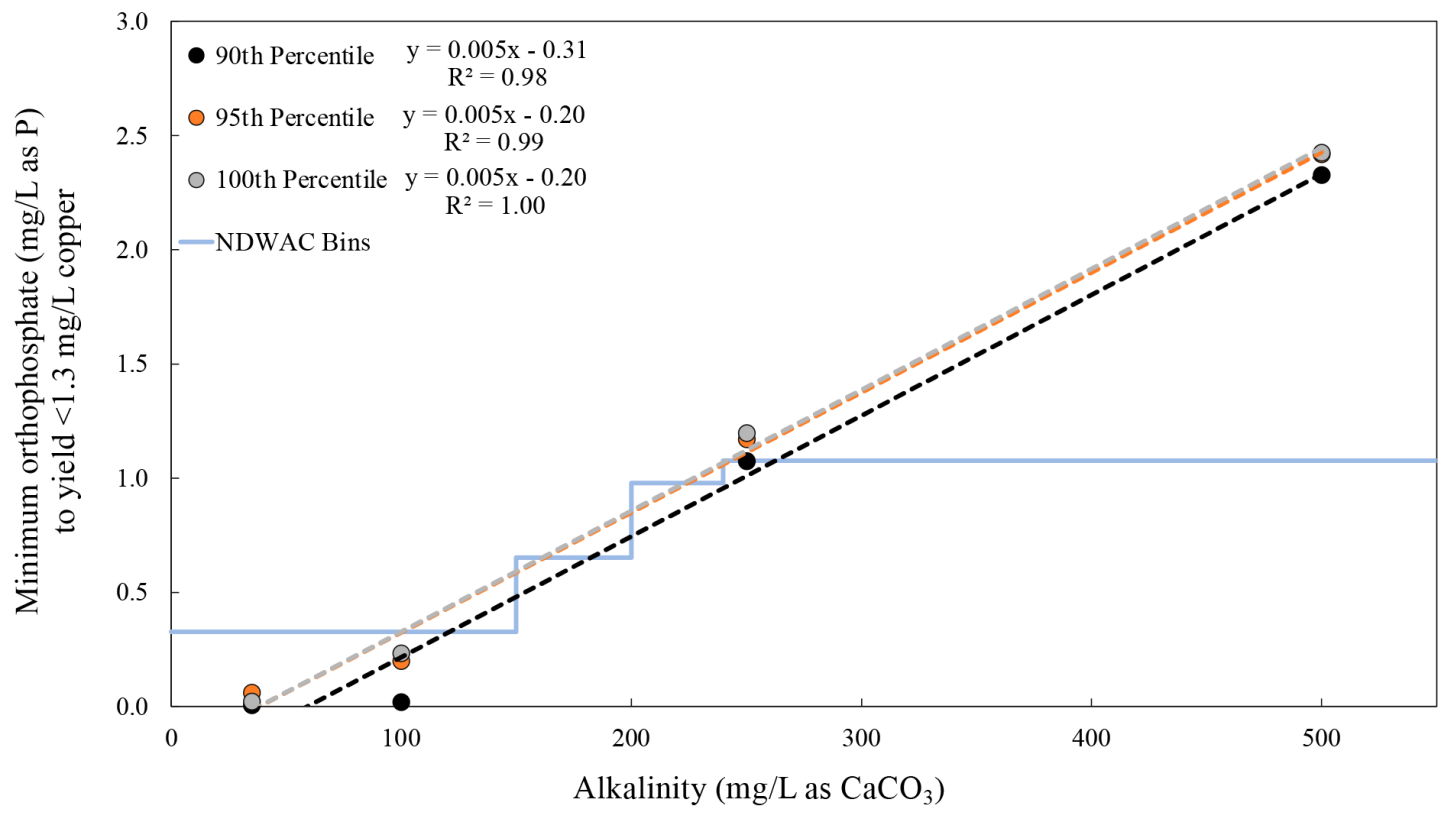
Linear regressions defining the “minimum” orthophosphate criteria for varying alkalinities to define waters “non-aggressive” to copper. Orthophosphate values represent the highest interpolated value, after either 4 or 22 weeks of testing, from linear correlations of orthophosphate and maximum (100th percentile), second (95th percentile) and third (90th percentile) highest copper concentration for each alkalinity tested. The bin system recommended by the NDWAC consensus statement is noted by the light blue line.^15^ Water conditions, including varying pH with alkalinity, are presented in Table 1.

The minimum orthophosphate doses that yielded copper below the 1.3 mg/L AL, which could serve as “Non-Aggressive Water Criteria” (Figure 1) were determined for each alkalinity (example method in section S1). To be conservative, the highest, second highest, and third highest copper concentrations (100th, 95th, and 90th percentiles) of the 20 pipe segments were utilized for determining orthophosphate criteria. Note that while this limited data interpretation is useful for comparison to compliance sampling values, it should not be construed to consider all sources of error that would be encountered at field sites. Data after four and 22 weeks of exposure for three orthophosphate doses were interpolated to determine the orthophosphate doses needed at each alkalinity to achieve copper below the 1.3 mg/L AL. “Worst case” linear regressions (Figure 3) were developed from interpolated results using the highest value after either 4 weeks or 22 weeks of pipe aging. Regressions demonstrated good correlations (*R*^2^ > 0.98) and generally good agreement between 90th, 95th and 100th percentile regressions. Results verify that increasing orthophosphate doses are required to reduce cuprosolvency at higher alkalinities as predicted by Schock et al.^1,20^

These regressions provide a conservative and continuous method to help utilities estimate required orthophosphate doses needed to control cuprosolvency as a function of alkalinity, under what are predicted to be relatively aggressive water chemistry conditions in terms of sulfate and NOM.^6,9,16,24,27,33,34^ Results also demonstrate relatively good agreement with the specific minimum orthophosphate levels tentatively recommended by the NDAWC consensus statement (Figure 3).^15^ However, for the water chemistries tested, the NDWAC bins may not be conservative enough to control the cuprosolvency for higher alkalinity waters within each bin. This highlights the value of the continuous regression approach for more precise estimation of “minimum” orthophosphate doses needed based on a water’s maximum alkalinity to avoid situations with sustained high cuprosolvency.

Our results suggest a linear correlation (*R*^2^ > 0.98) between alkalinity and the minimum orthophosphate dose within the range of water conditions tested. Modeling of copper solubility at equilibrium with Cu_3_(PO_4_)_2_ solid using Mineql+ also indicated a quasi-linear relationship at a given pH, with model predictions deviating from linear regressions by 16% or less (pH 7.25 and 8, and alkalinities >100 to 500 mg/L as CaCO_3_) (Figure S7). However, when considering Mineql+ model predictions corresponding to water conditions used to develop “minimum” orthophosphate criteria, the Mineql+ model had a slope over 4 times higher than that of the “minimum” OP linear regressions developed by the experiment herein (Figure S8). Other literature also notes inaccuracies in predicting soluble copper based on orthophosphate dose from chemical equilibrium modeling. In response, Lytle et al. developed an empirical model, which fit data from a fresh-solids cuprosolvency method.^36^ That model predicts much lower residual orthophosphate doses than those identified from our “minimum” orthophosphate criteria developed from pipe testing, even with our much longer experimental duration of 5.5 months vs their equilibration time of 10 min.

### 3.2 Development of a Simplified Fresh Solid Cuprosolvency Test Method

A cuprosolvency test method was developed using fresh copper solids. Although cuprosolvency tests using fresh solids may pose advantages, there were several complications that precluded their use for developing analogous minimum orthophosphate dose criteria as developed using copper tube in the previous section. Specifically, in initial tests, when only a single dose was used, the orthophosphate was consumed, and the orthophosphate residual that controls solubility varied. Two modified methods were used to maintain higher phosphate residuals during the test.

#### 3.2.1 Comparison of Cuprosolvency Testing Methods

Simplified bench cuprosolvency testing, which starts with fresh cupric solids, may pose several advantages over testing using copper tube. In new pipes, cupric hydroxide solids are thought to form from cuprous solids over a period of days, weeks, or months, so it could take time for such solids to accumulate and control copper dissolution.^6,16^ Once they form, these cupric hydroxides can then begin the aging process. Starting the testing with fresh Cu(OH)_2_ particles, would possibly accelerate that process, allowing solids aging to be observed more quickly, with 6.7 times less effort, 30% less initial cost, and up to 7 times lower cost for subsequent tests than in copper tube tests (Table 2, section S3).^6,16^ However, this study demonstrates that fresh-solids approaches require further development to overcome several potential limitations. Finally, although in-home testing may be the least costly approach for utilities to detect cuprosolvency problems on a per home basis, it may require testing of many homes and coordination with homeowners to demonstrate that cuprosolvency is not a problem, in which case an alternative approach using the fresh solids method would be of value.

**Table 2:** Comparison of Various Cuprosolvency Test Methods^a^

	Materials				Time			Overall		
Appartatus	Initial Materials	Consumables	Initial Materials Cost	Consumable Cost	Required Tasks	Labor	Labor Costs	Duration	Cost For Initial Test	Cost For Subsequent Tests
Fresh Solids Test	Carboy, bottles, Stir plate	Reagents, syringes, filters, analysis	$1,100	$1,600	Preparing water and particles, sampling, sample analysis	12 h.	$240	30 days	$2,900	$370
Copper Tube Test	Carboy, Temperature Control, Weighted lid, tray, bottles, pipe reamer	Reagents, stoppers, pipe segments, rubber bands labels, plastic wrap, cups, analysis	$1,400	$1,100	Preparing pipes, water changes, sampling, sample analysis	80 h.	$1,600	6 months	$4,200	$2,600
In-Home Test	Bottles	Reagents, analysis	$80	$200	On-site sampling, sample analysis	6 h./visit	$120	1 week	$400	$180

a Assumes access to ICP-MS/MS ($6 per sample), pH meter, and wages of $20 per hour. Cost estimates, detailed in section S3, are on per-home basis or for initial experiment. Costs for subsequent tests account for the initial materials and extra consumables that could be used in additional experiments.

#### 3.2.2 Initial Testing Using Fresh Copper Solids

**Figure 4:**
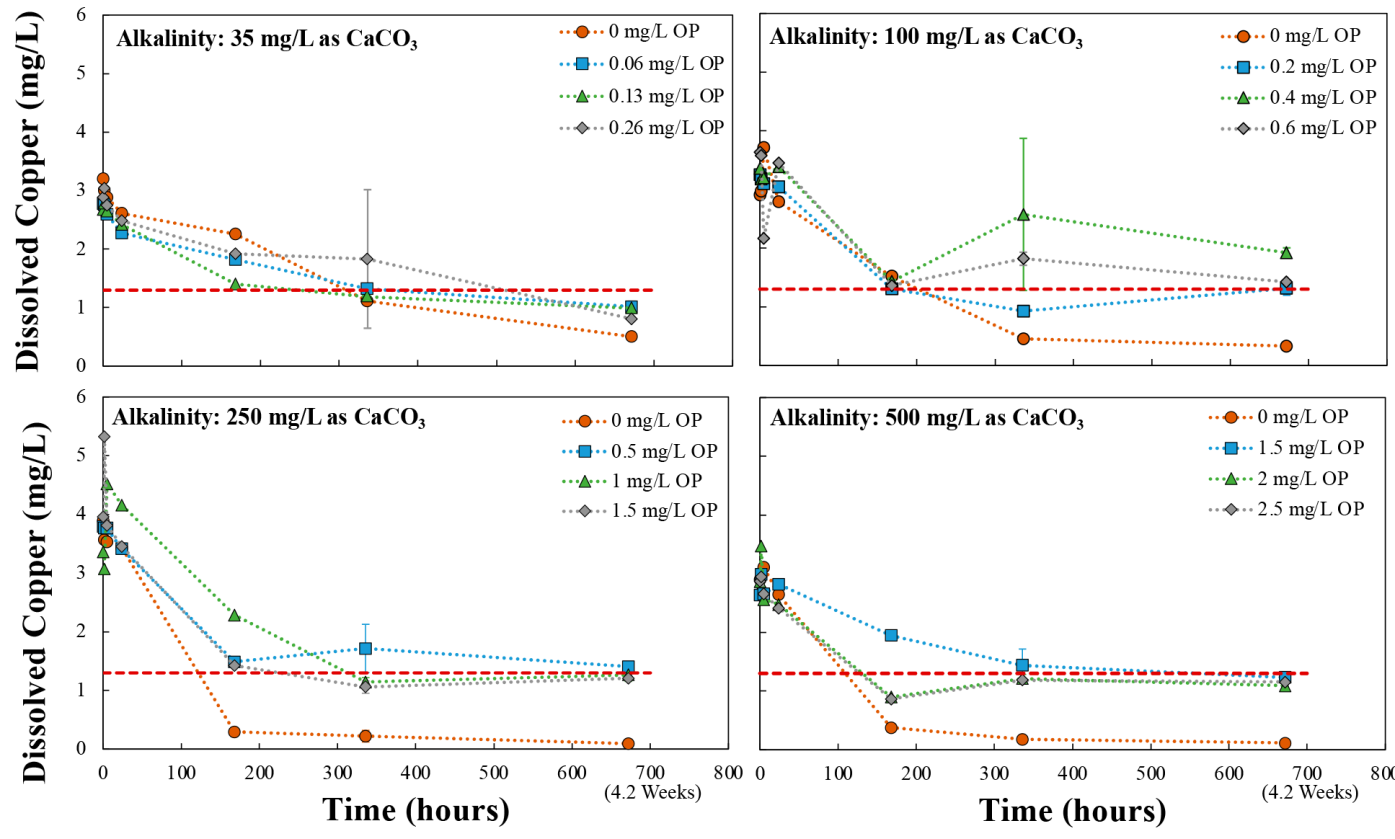
Mean dissolved copper release from copper particles treated with varying levels of orthophosphate corrosion control (*n* = 5 for weeks 2 and 4; *n* = 1 for all other times). Red dotted lines denote the 1.3 mg/L action level. All orthophosphate (OP) doses are in mg/L as P.

Initial cuprosolvency tests using fresh copper solids were tested using slight modifications to a prior simple method.^5^ Results indicated that aging and scale formation may progress faster than in tube-based tests. In tests with fresh solids, copper concentrations fell well below the AL (0.09 to 0.50 mg/L dissolved copper) within 1 month (Figure 4) for all alkalinity conditions without orthophosphate but were consistently higher in tube experiments even after 35 weeks of aging (0.65 to 2.50 mg/L after 1 month, 0.43 to 1.35 mg/L after 35 weeks). This indicates that particle-based methods may not be indicative of short-term cuprosolvency behavior in new pipe, which can cause acute health concerns, but may be more representative of the final mineral product achieved with long-term aging. It is unclear how long it would take to achieve these longer-term results in copper tube testing with regular water changes. Therefore, particle tests may be particularly useful for evaluating scale formation in conditions where longer term scale transitions may be expected, as opposed to orthophosphate treated waters, where scales form relatively quickly.

One challenge of applying short-term simple cuprosolvency tests in the presence of phosphate inhibitors is simulating orthophosphate residuals observed in copper pipes during stagnation in real systems. Specifically, in new pipes, orthophosphate is depleted from the water as it is incorporated into pipe scale, whereas upon further flow and scale accumulation, orthophosphate is not significantly depleted during stagnation. For instance, in copper tube experiments with orthophosphate replenishment via water changes, 16–32% of orthophosphate was taken up in 100 mg/L as CaCO_3_ alkalinity waters at the end of the first week of testing, but only up to 11% was taken up after 3 weeks of testing. In fresh solid tests without orthophosphate replenishment, 65–98% of the orthophosphate was consumed by the end of the 1-month experiment. Because orthophosphate residuals could not be maintained, results from fresh-solids tests were not comparable to copper tube tests and were not suitable for determining “minimum” orthophosphate criteria analogous to those presented for copper tube tests (Figure 3). Further, the fresh solid tests also cannot capture manufacturer differences observed in tests with real tubes.

Thus, in the initial fresh solids protocol without replenishing orthophosphate, cuprosolvency was an average of 0.42 mg/L higher (difference 0.12 to 1.22 mg/L, range 0.81 to 1.92 mg/L) than in corresponding copper tube tests (22 weeks, range 0.62 to 1.06 mg/L). It was nonetheless considered promising even when underdosing phosphate that, analogous to copper tube testing, orthophosphate had relatively little effect on cuprosolvency at low alkalinity and yielded higher long-term cuprosolvency than conditions without orthophosphate at high alkalinity. These results are consistent with the short-term benefit and long-term detriment resulting from the formation of higher solubility cupric phosphate scales.^1,6,7,9−11,16−19,31,32,39,40^

#### 3.2.3 Modified Cuprosolvency Test Using Fresh Copper Solids

The cuprosolvency method using fresh copper solids was modified to overcome the orthophosphate demand of the aging solids. The modified method utilized a 1 L batch of test water (pH 7.5, 250 mg/L as CaCO_3_ alkalinity, 0.2 mg/L NOM, and 200 mg/L sulfate) and either one initial 4.3 mg/L as P dose of orthophosphate or the same total orthophosphate added throughout the test to maintain a residual of about 1.5 mg/L as P. These dosing strategies yielded variable cuprosolvency results in comparison to previous cuprosolvency test methods.

Results from successive dosing experiments (section S4) demonstrate initial depletion of orthophosphate coupled with reductions in soluble copper (Figure S13), as may be expected if cupric phosphate solids rapidly formed and controlled cuprosolvency.^1,6^ Further, dissolved copper concentrations stabilized much faster at a concentration just over half that observed in tube experiments with similar conditions, potentially indicating more complete cupric phosphate scale formation. In contrast, when the same total orthophosphate was added as one initial dose, residual orthophosphate quickly fell below the 1.5 mg/L as P target, yielding almost three times higher copper (1.41 mg/L dissolved copper, 5 h) than when the target residual was maintained, potentially indicating differences in particle and/or scale formation. Overall, these results demonstrate that the timing and dose of orthophosphate addition can affect particle formation via orthophosphate uptake and cuprosolvency. Further, they highlight the need for additional method development if it is desired to achieve a standardized particle cuprosolvency test that accounts for factors such as solution mixing while maintaining a target residual.

## 4 Implications

Although the gaps in copper corrosion control were not addressed in the recent LCR revisions,^29,30^ proactive utilities may choose to address sustained elevated cuprosolvency to protect public health or alleviate consumer complaints. For example, it may be relatively easy for proactive utilities to add copper tube testing, to standard CCT optimization studies with lead pipe loops or bench testing.^42^ This work expands upon the NDWAC recommended guidance to help utilities predict the orthophosphate doses needed to address sustained elevated cuprosolvency in their distribution systems. Utility use of this framework, as well as the development of faster and simpler, fresh solid cuprosolvency test methods, could help to quickly identify water conditions likely to yield “low cuprosolvency” water without costly in-home testing, potentially saving utilities time and resources. However, caution should be used in applying fresh solids tests until further study can confirm which solids formed and overcome the challenges with fresh solids tests that were demonstrated in this study. Although results indicate that orthophosphate may not be necessary to control cuprosolvency in the short term in lower alkalinity waters or even in certain higher alkalinity waters that age to lower solubility solids, regressions were developed to help determine orthophosphate doses needed for circumstances where cuprosolvency is problematic. One advantage of this “minimum” orthophosphate approach is that the systems reached a pseudosteady state relatively quickly, indicating that utilities can expect to see a characteristic response within weeks of orthophosphate addition.

It is also important to note that orthophosphate addition for corrosion control can have multiple benefits. First, orthophosphate is often added to control corrosion of lead and iron, in which case its effect on copper is a side benefit if it reduces cuprosolvency, or a detriment if it increases cuprosolvency.^1,9,31,32^ Orthophosphate addition can be expected to be effective even in higher alkalinity waters without the potential for causing precipitation of carbonate scales that would arise from raising the pH.^31^ In fact, in those situations, orthophosphate can reduce the scaling potential of the water.^43^

Finally, orthophosphate treatment yields nearly immediate reductions in cuprosolvency, with low and relatively stable copper concentrations possible after less than 1 week of treatment, which could be particularly beneficial for utilities experiencing abrupt changes in the water source or quality. However, the use of orthophosphates also has certain drawbacks. For example, continuous orthophosphate addition is required to maintain cuprosolvency reductions, with higher concentrations sometimes needed to control cuprosolvency than to control lead release.^9,20^ These high orthophosphate doses could make it difficult for utilities to effectively control cuprosolvency while meeting wastewater phosphate discharge limits and pose concerns about expense and sustainability of phosphate use.^9,11^ Further, caution should be used when applying this relatively simplistic approach since the effectiveness of orthophosphate treatment and dose may vary with water quality, including pH, alkalinity, and other parameters, sometimes in unanticipated ways.^1,20,24,28,40^ As observed in this study, higher orthophosphate doses are required for higher alkalinity to achieve cuprosolvency reductions. Finally, this study confirms that, in some cases, despite the initial benefit, orthophosphate use can lead to higher long-term cuprosolvency than conditions where pipes age normally and form lower solubility solids.^1,6,7,9−11,16−19,31,32,39,40^

More research could improve the practice of optimizing orthophosphate doses. For example, further mechanistic study, investigation of thermodynamic data, and research on spectroscopic phase identification for X-ray amorphous solids for various orthophosphate minerals would aid in understanding and modeling orthophosphate effectiveness and doses needed.^1^ Very high levels of NOM have been reported to reduce the efficiency of orthophosphate corrosion inhibitors, indicating that higher orthophosphate doses may be needed in some situations for cuprosolvency control.^9,44^ Further, the effectiveness of blended phosphates or polyphosphates for cuprosolvency control warrants further investigation, as they have been suggested to complex copper and were associated with sustained elevated copper above the AL, even while the utility was in compliance with the LCR.^20,45^ An improved cuprosolvency test using fresh copper solids could be helpful for more quickly evaluating the effects of these parameters; however, it requires further development to overcome challenges maintaining constant residual concentrations to mimic the replenishment of constituents as occurs in pipe systems with flowing water. Finally, it would also be beneficial to determine the “minimum” pH vs alkalinity regression criteria, suggested by the NDWAC, which would allow utilities to simultaneously consider either orthophosphate or pH/alkalinity control methods.

## Supporting Information Available

Method for determination of “minimum” orthophosphate doses and regression criteria; Mineql+ modeling of cuprosolvency and comparisons of “minimum” orthophosphate criteria with various models; estimation of cuprosolvency testing costs; observed differences in cuprosolvency based on tube manufacturer; and cuprosolvency testing results using fresh copper solids. (PDF)
