# Supplementary material for: Considering a Utility-Centric Framework Based on “Minimum Orthophosphate” Criteria for Mitigation of Elevated Cuprosolvency in Drinking Water
Source: Environ Sci Technol. 2024 Mar 12;58(12):5606–15. doi: 10.1021/acs.est.4c00583 (PMC10976879; doi:10.1021/acs.est.4c00583)
Supplement: Supplementary file 1 — es4c00583_si_001.pdf [file es4c00583_si_001.pdf]

Supplemental Information

"Considering a Utility-Centric Framework based on 'Minimum Orthophosphate' Criteria for Mitigation of Elevated Cuprosolvency in Drinking Water"

Rebecca B. Kriss[1], [a]Emily Smith[1], [a]Grace Byrd[1], Michael Schock[2], Marc A. Edwards[1*]

[a]E.S and G.B contributed equally to this paper.
[1] Virginia Polytechnic and State University, Civil and Environmental Engineering, 418 Durham Hall, Blacksburg, VA 24061
[2] Retired, Cincinnati, OH 45230

*Corresponding author: Marc A. Edwards: edwardsm@vt.edu; Address: 407 Durham Hall 1145 Perry Street, Blacksburg, VA 24060

Description of Supporting Information (18 pages, 13 Figures)

Section S1. Determination of "minimum" orthophosphate criteria
Section S2. Mineql+ modeling of cuprosolvency
Section S3. Estimation of cuprosolvency testing costs
Section S4. Cuprosolvency testing using fresh copper solids
Figure S1. Minimum orthophosphate doses achieving copper below the Action Level for 100th percentile (Maximum) copper concentrations after 22 weeks of pipe aging.
Figure S2. Minimum orthophosphate doses achieving copper below the Action Level for 95th percentile (second highest) copper concentrations after 22 weeks of pipe aging.
Figure S3. Minimum orthophosphate doses achieving copper below the Action Level for 90th percentile (third highest) copper concentrations after 22 weeks of pipe aging.
Figure S4. Minimum orthophosphate doses achieving copper below the Action Level for 100th percentile (Maximum) copper concentrations after 4 weeks of pipe aging.
Figure S5. Minimum orthophosphate doses achieving copper below the Action Level for 95th percentile (second highest) copper concentrations after 4 weeks of pipe aging.
Figure S6. Minimum orthophosphate doses achieving copper below the Action Level for 90th percentile (third highest) copper concentrations after 4 weeks of pipe aging.
Figure S7. Comparison of "minimum" orthophosphate regressions with constant-pH models.
Figure S8. Comparison of "minimum" orthophosphate regressions with models with corresponding varying pH.
Figure S9. Observed differences in cuprosolvency based on tube manufacturer in waters with 35 mg/L as $CaCO_3$ alkalinity at pH 7.25.
Figure S10. Observed differences in cuprosolvency based on tube manufacturer in waters with 100 mg/L as $CaCO_3$ alkalinity at pH 7.25.
Figure S11. Observed differences in cuprosolvency based on tube manufacturer in waters with 250 mg/L as $CaCO_3$ alkalinity at pH 7.5.
Figure S12. Observed differences in cuprosolvency based on tube manufacturer in waters with 500 mg/L as $CaCO_3$ alkalinity at pH 8.
Figure S13. Dissolved copper release from particles treated with 4.3 mg/L as P orthophosphate added all at once or replenished to a constant residual concentration.

Section S1. Determination of “minimum” orthophosphate criteria

The minimum orthophosphate (OP) dose needed to achieve copper concentrations below the 1.3 mg/L action level were estimated based on water alkalinity. To determine this, three orthophosphate doses were tested for each of four alkalinities (n= 20 pipe segments per condition).

Results for the 90th, 95th and 100th percentile (18th, 19th, and 20th highest) copper values after 4 and 22 weeks of tube/pipe conditioning (8 weeks with water changes and a 14-week extended stagnation period) were interpolated for each alkalinity to determine the minimum OP dose that yielded copper below the action level. An example interpolation for the 100th percentile (maximum copper value) results after 22 weeks of pipe aging is presented in **Figure S1**. Additional interpolations are presented for the 90th, 95th and 100th percentile data after 4 and 22 weeks of pipe aging in **Figures S2-S6**.

Linear regressions of the “minimum” OP dose needed to get copper below the action level were developed based on the water’s alkalinity and “worst case” OP values. To simulate the “worst case” scenario, the higher interpolated orthophosphate value after either week 4 or week 22 was used for each alkalinity to construct the linear regressions. Regressions are provided for the 90th, 95th and 100th percentile data (**Figure 3).**

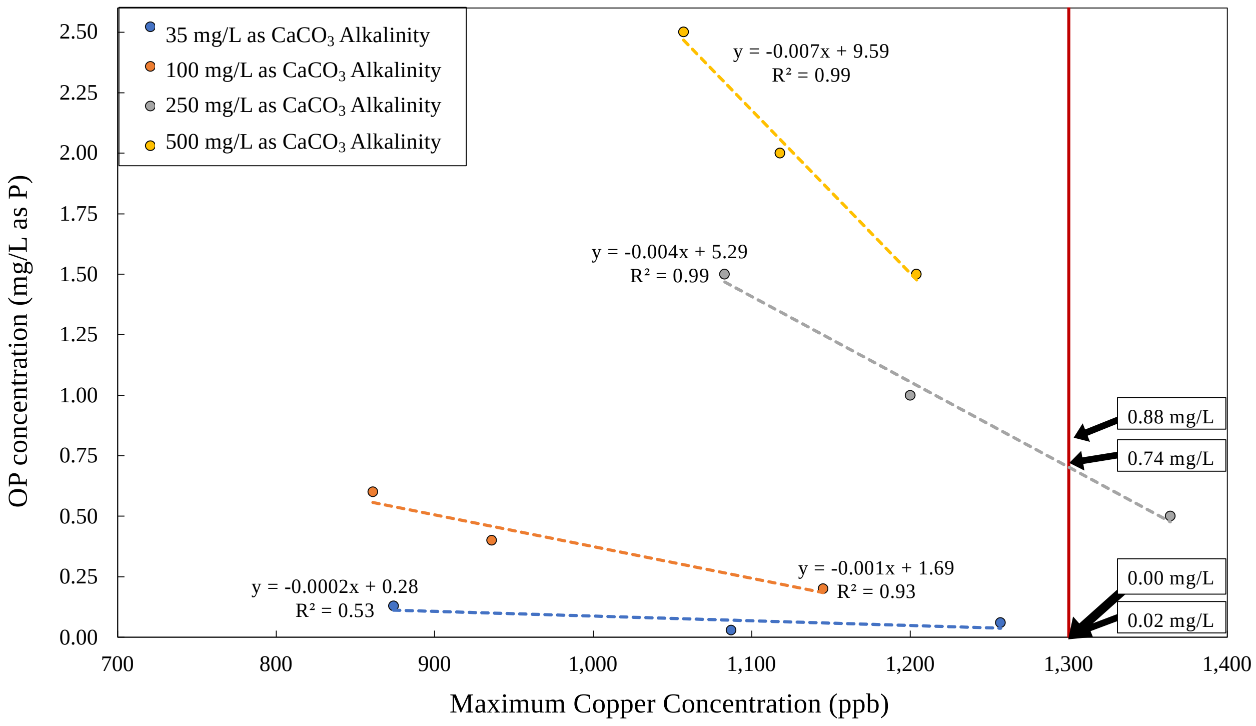

Figure S1. Illustrative graph for defining minimum orthophosphate dose to achieve copper concentrations below the Action Level (solid red line) for the 100th percentile (Maximum) copper concentrations after 22 weeks of pipe aging. Arrows with boxes denote the “minimum” orthophosphate (mg/L as P) predicted by the regressions to yield copper below the action level.

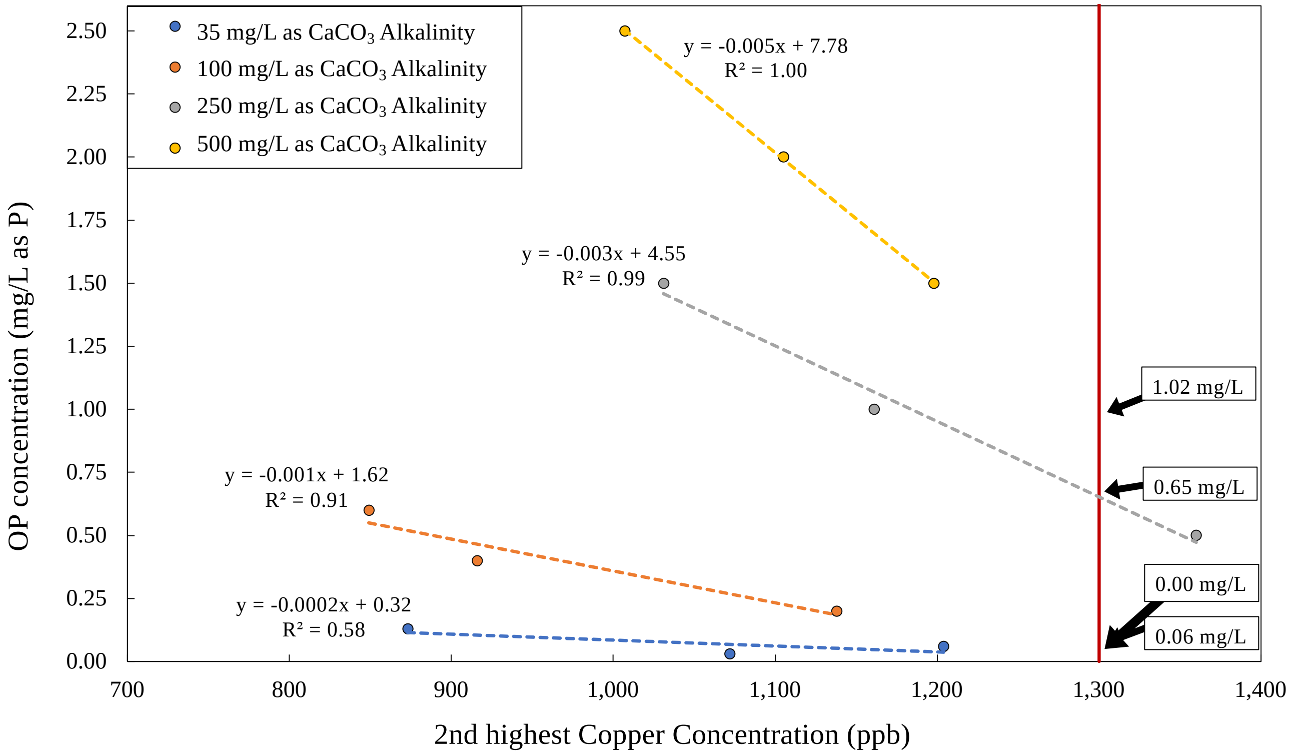

Figure S2. Illustrative graph for defining minimum orthophosphate dose to achieve copper concentrations below the Action Level (solid red line) for the 95th percentile (second highest) copper concentrations after 22 weeks of pipe aging. Arrows with boxes denote the "minimum" orthophosphate (mg/L as P) predicted by the regressions to yield copper below the action level.

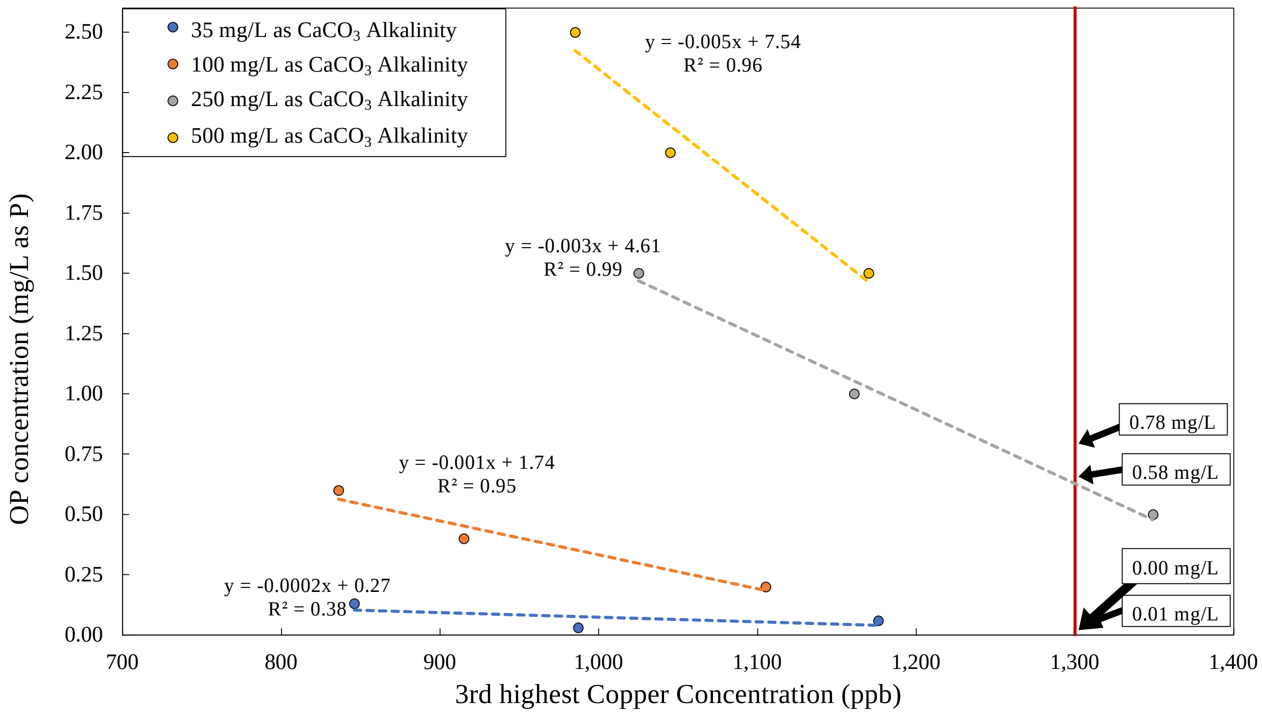

Figure S3. Illustrative graph for defining minimum orthophosphate dose to achieve copper concentrations below the Action Level (solid red line) for the 90th percentile (third highest) copper concentrations after 22 weeks of pipe aging. Arrows with boxes denote the “minimum” orthophosphate (mg/L as P) predicted by the regressions to yield copper below the action level.

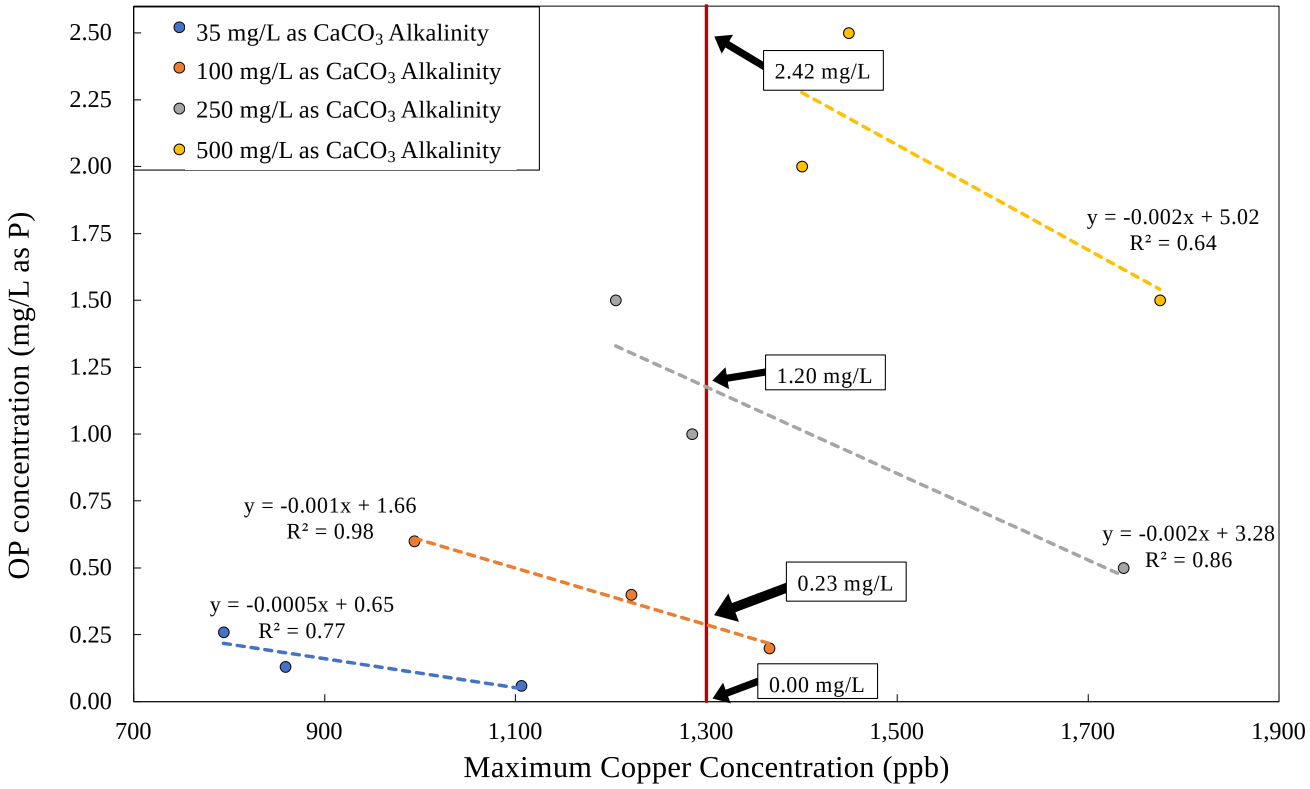

Figure S4. Illustrative graph for defining minimum orthophosphate dose to achieve copper concentrations below the Action Level (solid red line) for the 100th percentile (Maximum) copper concentrations after 4 weeks of pipe aging. Arrows with boxes denote the “minimum” orthophosphate (mg/L as P) predicted by the regressions to yield copper below the action level.

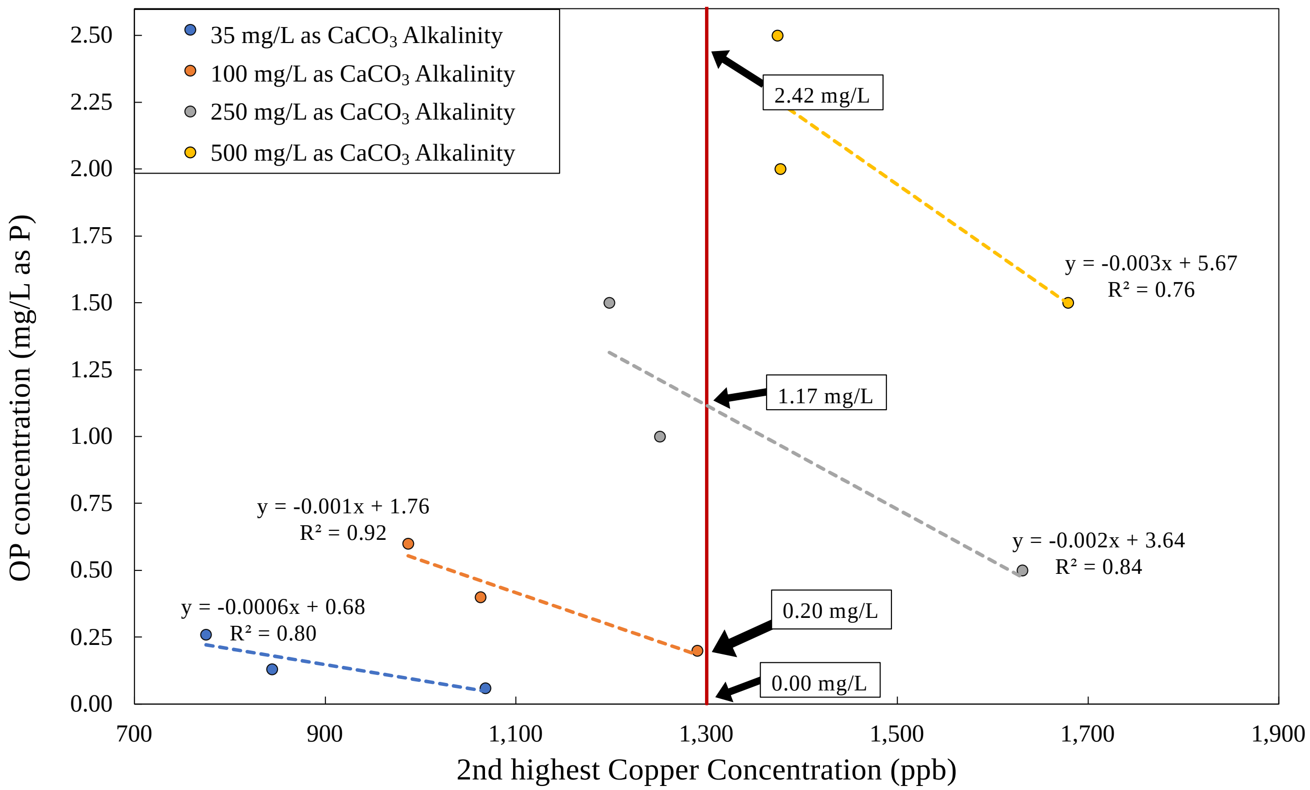

Figure S5. Illustrative graph for defining minimum orthophosphate dose to achieve copper concentrations below the Action Level (solid red line) for the 95th percentile (second highest) copper concentrations after 4 weeks of pipe aging. Arrows with boxes denote the “minimum” orthophosphate (mg/L as P) predicted by the regressions to yield copper below the action level.

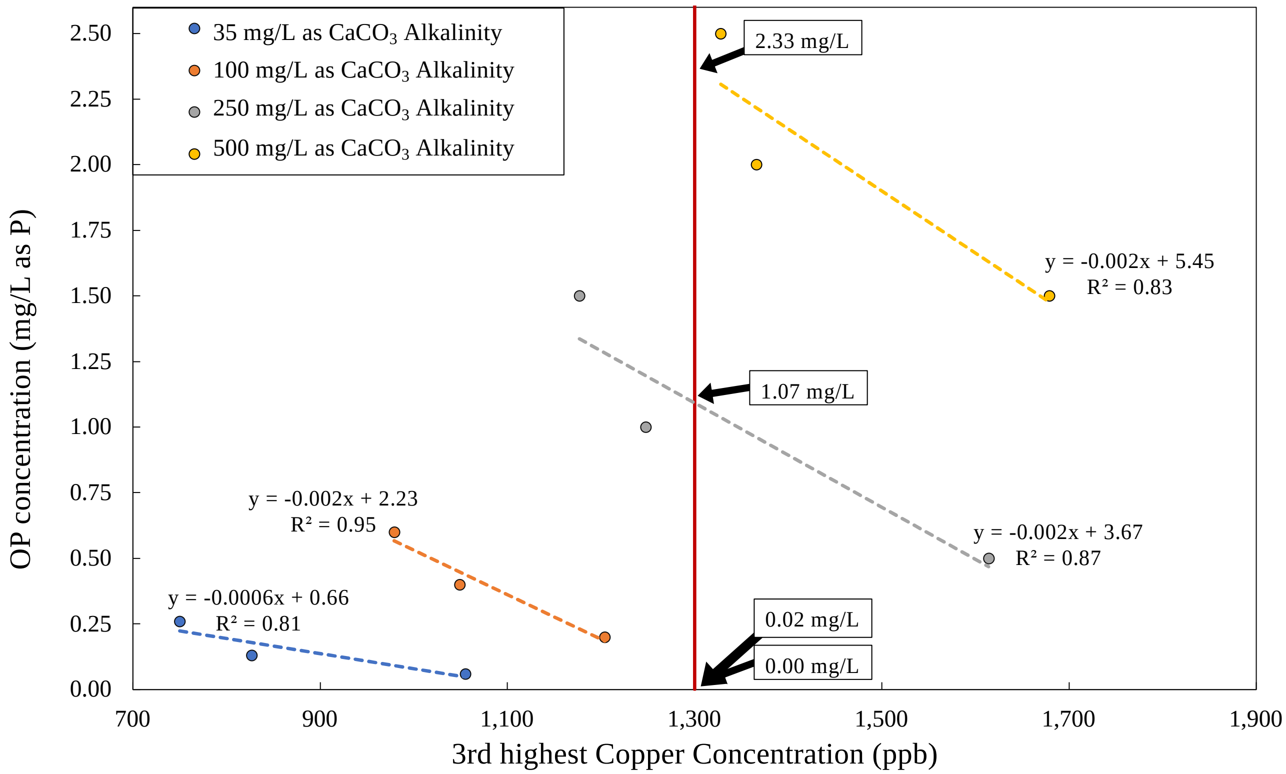

Figure S6. Illustrative graph for defining minimum orthophosphate dose to achieve copper concentrations below the Action Level (solid red line) for the 90th percentile (third highest) copper concentrations after 4 weeks of pipe aging. Arrows with boxes denote the "minimum" orthophosphate (mg/L as P) predicted by the regressions to yield copper below the action level.

Section S2. Mineql+ modeling of cuprosolvency

Cuprosolvency modeling was carried out using Mineql+ software (version 4.6) and assuming the system was in equilibrium with $Cu_3(PO_4)_2$ solid (13 °C). First, we calibrated the model to our data by adjusting the logK to fit our data at pH 7.5 and 100 mg/L as $CaCO_3$ alkalinity. The rest of the Mineql+ solubility models were run using the adjusted logK (37.6). Titration mode was used for each pH and alkalinity to determine the soluble orthophosphate residual concentration that would be needed to yield a soluble copper concentration of 1.3 mg/L.

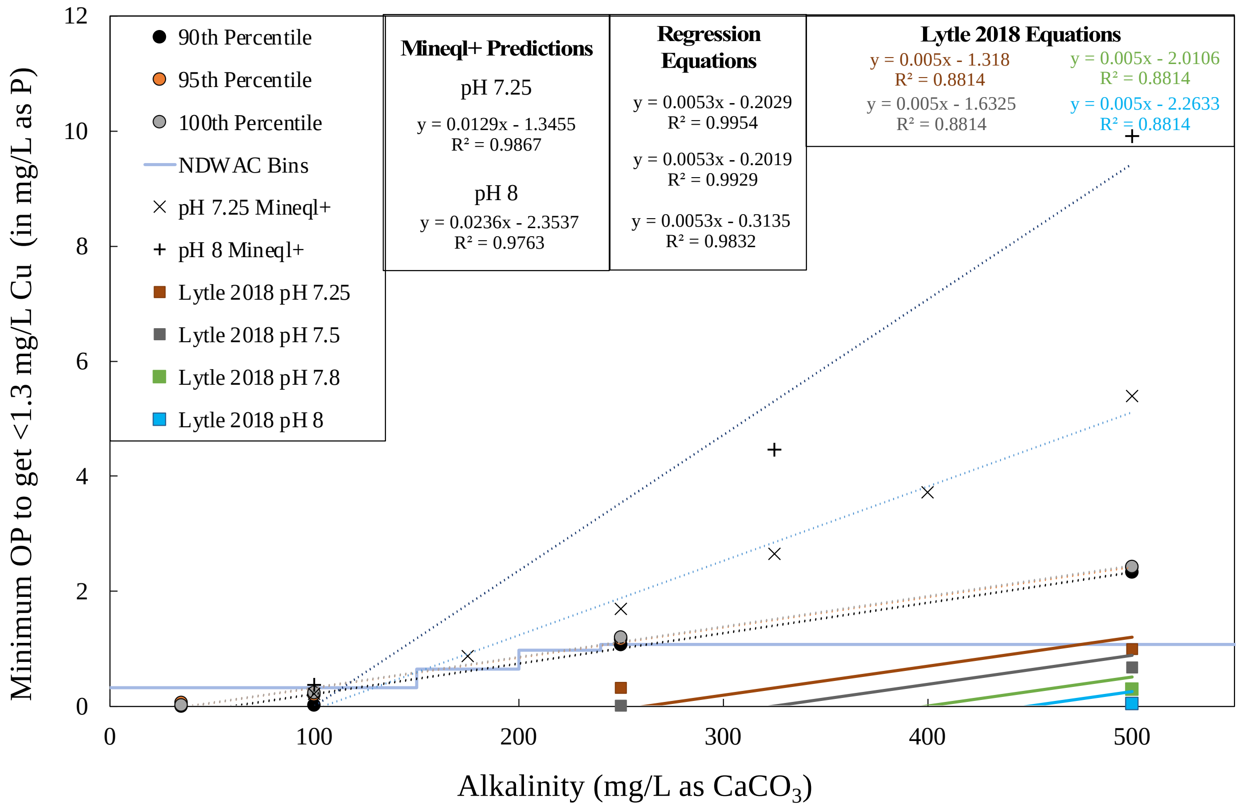

Figure S7. Comparison of linear regressions defining the "minimum" orthophosphate criteria for varying alkalinities and pH values to define waters as "non-aggressive" to copper with several constant-pH models. Orthophosphate values in the "minimum" orthophosphate criteria represent the highest interpolated value, after either 4 or 22 weeks of testing, from linear correlations of orthophosphate and maximum (100th percentile), second (95th percentile) and third (90th percentile) highest copper concentration for each alkalinity tested. Additional models presented include Mineql+ predictions at constant pH 7.25 and 8 for cuprosolvency in equilibrium with $Cu_3(PO_4)_2$ solid as well as empirical predictions based on Lytle et al.,[1] at constant pH 7.25, 7.5, 7.8, and 8.

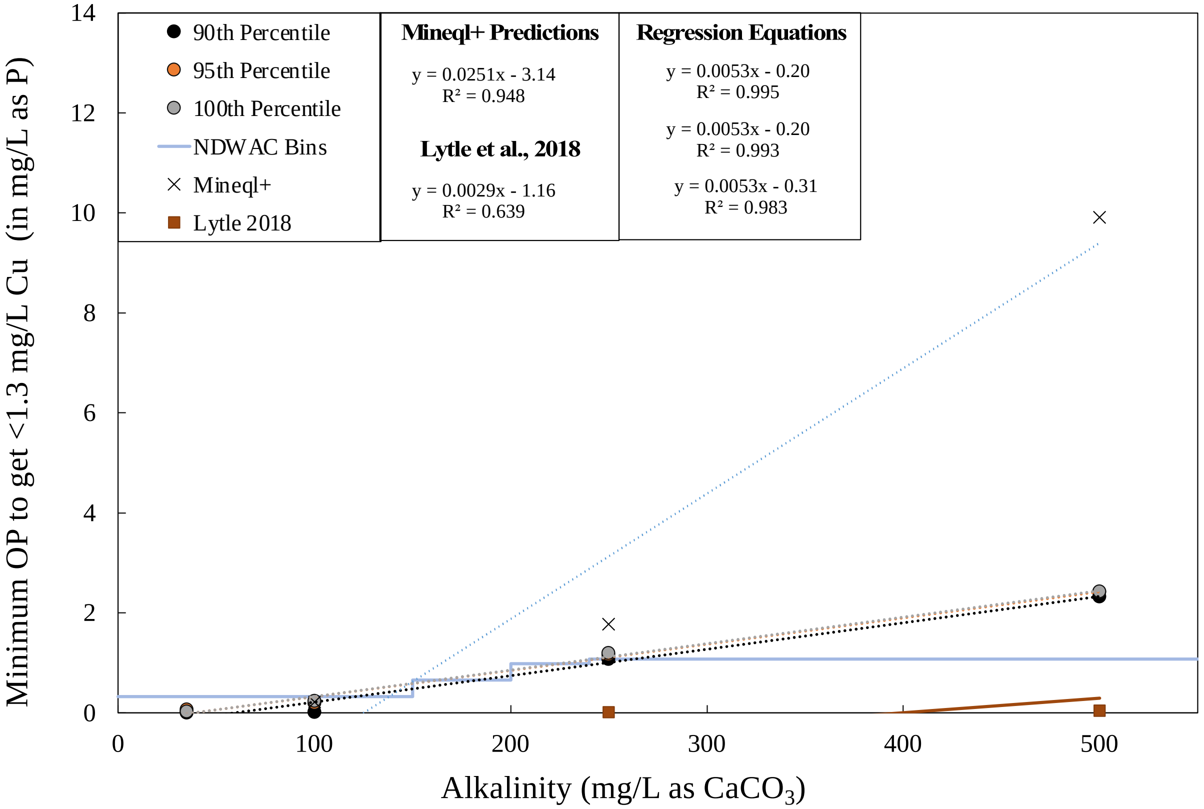

Figure S8. Comparison of linear regressions defining the "minimum" orthophosphate criteria for varying alkalinities and pH values to define waters as "non-aggressive" to copper with several models with corresponding varying pH. Orthophosphate values in the "minimum" orthophosphate criteria represent the highest interpolated value, after either 4 or 22 weeks of testing, from linear correlations of orthophosphate and maximum (100th percentile), second (95th percentile) and third (90th percentile) highest copper concentration for each alkalinity tested. Additional models presented include Mineql+ predictions based on equilibrium with $Cu_3(PO_4)_2$ solid and empirical predictions based on Lytle et al.,[1] with varying pH corresponding to those used to develop "minimum" orthophosphate criteria.

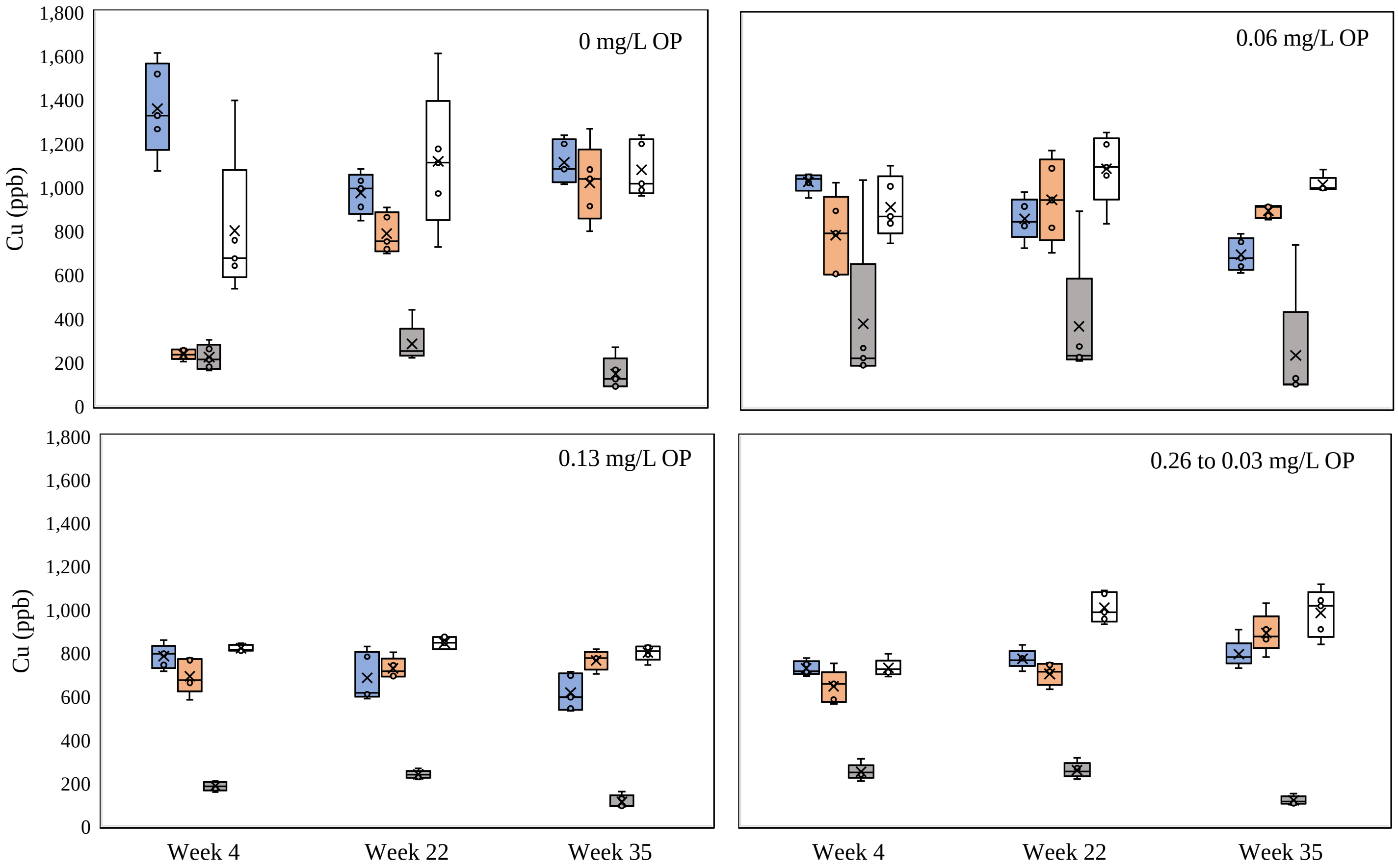

Figure S9. Box and whisker plots depicting observed differences in cuprosolvency based on tube manufacturer in waters with 35 mg/L as $CaCO_3$ alkalinity at pH 7.25 and varying orthophosphate doses after either 4, 22, or 35 weeks of pipe aging (n=5 tubes per manufacturer per water condition). All orthophosphate (OP) doses are in mg/L as P. Copper tube brands are represented as follows: Brand A is blue, Brand B is orange, Brand C is grey, and Brand D is white, which correspond to the brands in a just published companion study (Kriss and Edwards, 2023).[2]

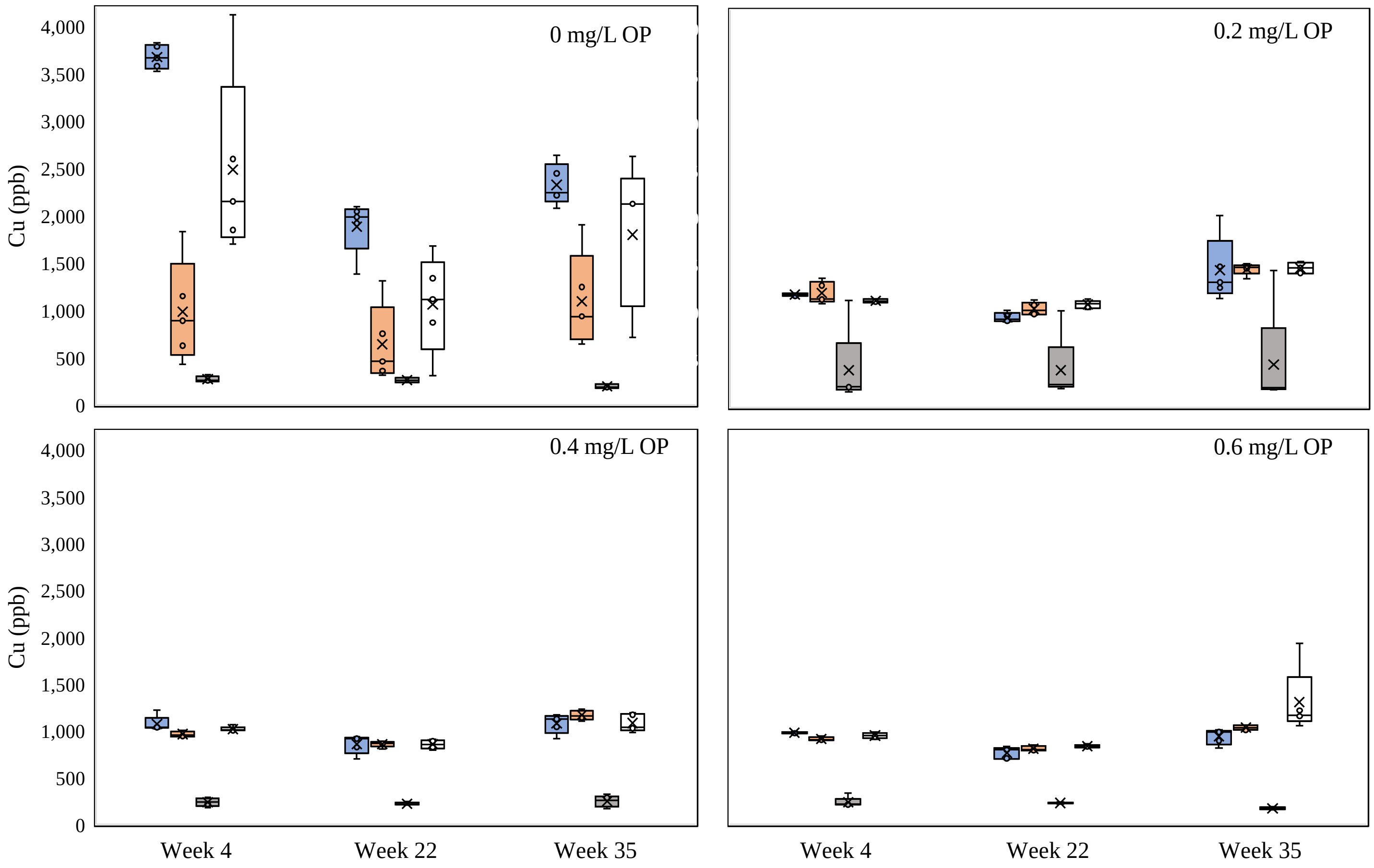

Figure S10. Box and whisker plots depicting observed differences in cuprosolvency based on tube manufacturer in waters with 100 mg/L as $CaCO_3$ alkalinity at pH 7.25 and varying orthophosphate doses after either 4, 22, or 35 weeks of pipe aging (n=5 tubes per manufacturer per water condition). All orthophosphate (OP) doses are in mg/L as P. Copper tube brands are represented as follows: Brand A is blue, Brand B is orange, Brand C is grey, and Brand D is white, which correspond to the brands in a just published companion study (Kriss and Edwards, 2023).[2]

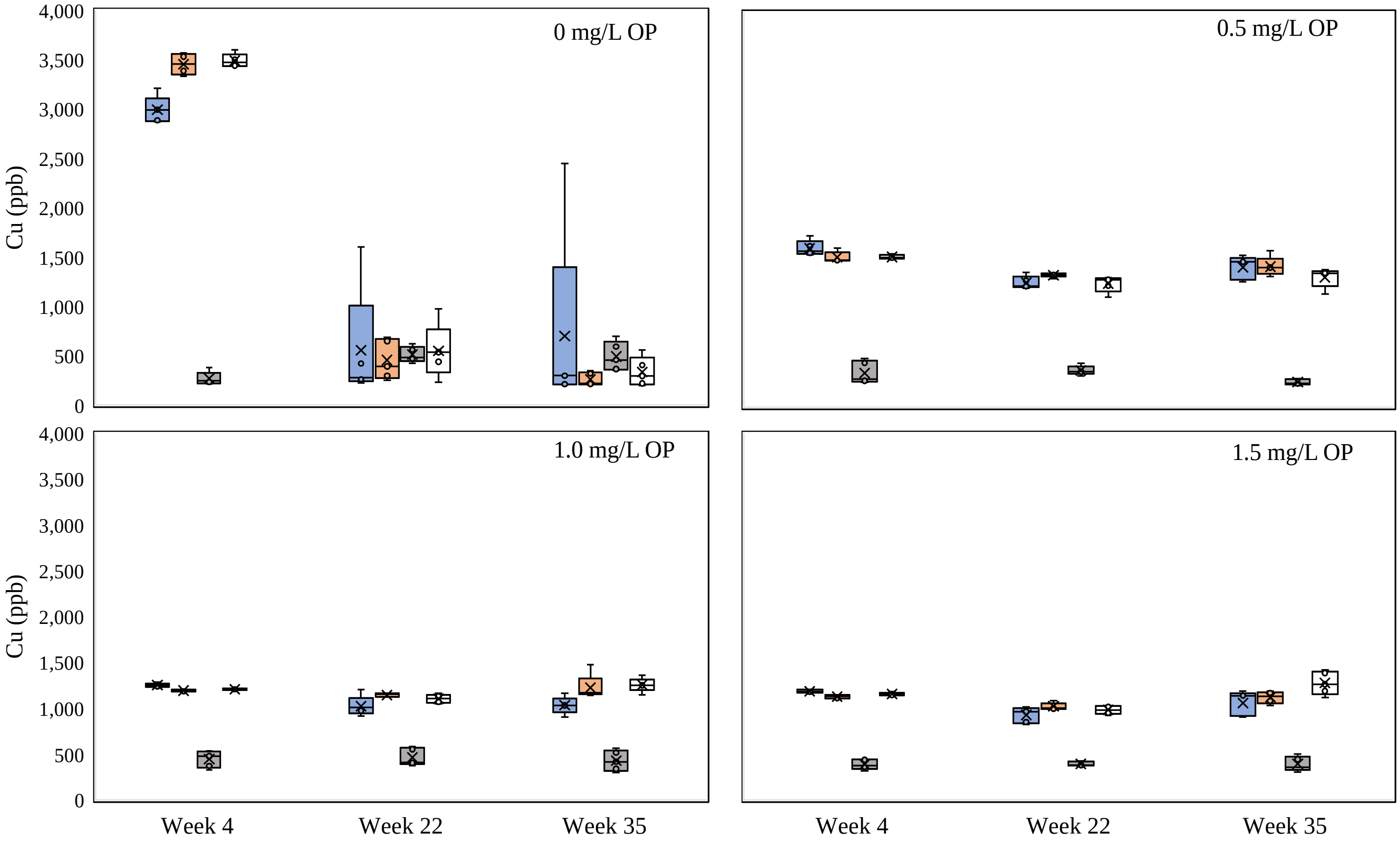

Figure S11. Box and whisker plots depicting observed differences in cuprosolvency based on tube manufacturer in waters with 250 mg/L as $CaCO_3$ alkalinity at pH 7.5 and varying orthophosphate doses after either 4, 22, or 35 weeks of pipe aging (n=5 tubes per manufacturer per water condition). All orthophosphate (OP) doses are in mg/L as P. Copper tube brands are represented as follows: Brand A is blue, Brand B is orange, Brand C is grey, and Brand D is white, which correspond to the brands in a just published companion study (Kriss and Edwards, 2023).[2]

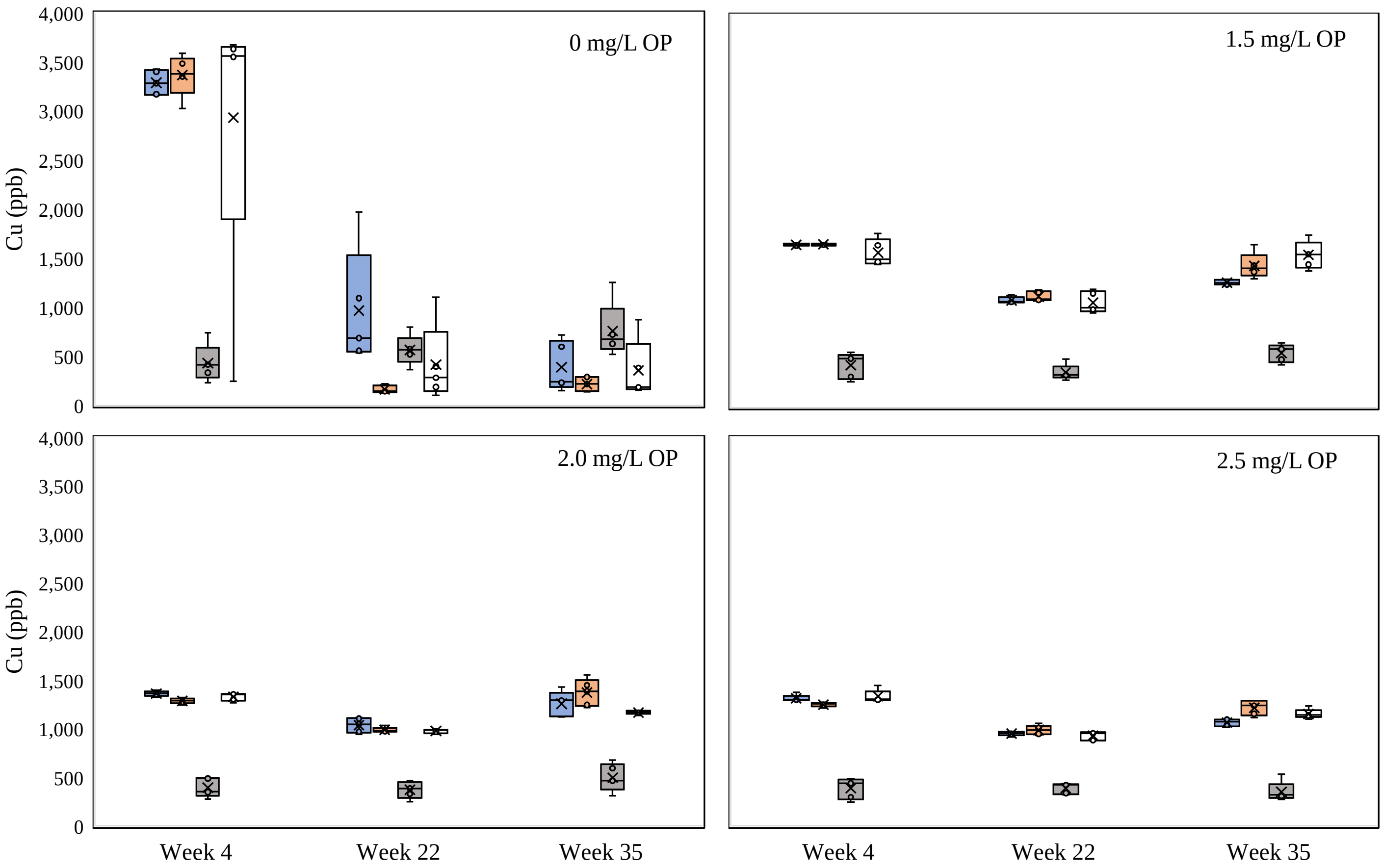

Figure S12. Box and whisker plots depicting observed differences in cuprosolvency based on tube manufacturer in waters with 500 mg/L as $CaCO_3$ alkalinity at pH 8 and varying orthophosphate doses after either 4, 22, or 35 weeks of pipe aging (n=5 tubes per manufacturer per water condition). All orthophosphate (OP) doses are in mg/L as P. Copper tube brands are represented as follows: Brand A is blue, Brand B is orange, Brand C is grey, and Brand D is white, which correspond to the brands in a just published companion study (Kriss and Edwards, 2023).[2]

Section S3. Estimation of cuprosolvency testing costs

Three cuprosolvency tests were compared based on cost and time investment. All estimates assume access to ICP/MS-MS capabilities ($6 per sample), access to a working pH meter, labor costs of $20/hr., and use of utility or residential test waters. Estimates for cuprosolvency tests using copper tube and fresh copper solids reflect approximate labor and expenses from test in our laboratory. All estimates are for materials from Fisher Scientific or Home Depot, unless otherwise noted. Many of these costs would be similar for other laboratories or utilities trying to carry out similar experiments, however, some costs may vary based on sample analysis costs and where materials are sourced from.

Estimates for tests using fresh copper solids include initial one-time costs such as buying a carboy (10L, $137), plastic bottles (125 mL, two 12 packs, $93), and a stir plate with rpm control (10.25”, $849), all of which could be reused in subsequent trials. However, many consumables would be needed including: reagents (trace metals grade nitric acid, 500 mL, $180), syringes (10 mL, 50 pack, $56), syringe filters (150 pack, 0.45 μm, $839), disc filters (100 pack, 0.45 μm, $345), and sample analysis ($6 per sample for ICP-MS/MS analysis). The experiment was estimated to take 12 non-consecutive hours for initiating the experiment and collecting and analyzing samples.

Estimates for cuprosolvency tests in copper tube include one-time costs for a carboy (10L, $137), plastic bottles (125 mL, two 72 packs, $470), a pipe reamer ($15), materials for a storage tray ($16) and weighted lid ($58, including foam mat, metal sheet, glue, and metal pieces for weight), and a wine refrigerator for temperature control ($729), all of which could be reused in subsequent trials. In addition, these tests require consumables including copper tube (2 10’ pieces cut into 8.5” segments, $50), silicone stoppers (Cole Parmer 13D, 2 10 packs, $42), reagents (trace metals grade nitric acid, 500 mL, $180), sample analysis ($6 per sample for ICP-MS/MS analysis), and additional supplies ($26, including cups, plastic wrap, foil, rubber bands, labels). Finally, the experiment was estimated to take 80 non-consecutive hours, including procuring materials, preparing pipes (cutting, reaming, and cleaning), water changes three times per week, weekly composite sampling, monthly sampling of individual pipes, and sample preparation and analysis.

Estimates for in-home testing assumes a visit by staff to a resident’s home to perform sampling using a 3-bottle sequential sampling approach. Costs could vary widely depending on whether residents could sample themselves, how often sampling took place, and for what duration. We assumed sampling one time with the sampling taking total of 6 hours of utility personnel time. This labor includes time related to transit and sample collection in the home as well as sample preparation and analysis. In addition, this sampling would require 3 1L bottles ($84) as well as reagents (trace metals grade nitric acid, 500 mL, $180) and sample analysis ($6 per sample for ICP-MS/MS analysis).

Estimates also determined the approximate cost of running additional tests. These estimates assumed the same labor and analysis costs as previous tests. To account for additional materials that may be left over from previous tests, these estimates do not include costs for syringes, syringe filters, disc filters, nitric acid, or cupric nitrate reagents. Further, these estimates include

an additional 2 hours of labor for In-Home and Fresh Solids Tests and an additional 5 hours of labor for Copper Tube Tests to account for disposing of samples and cleaning bottles for reuse.

Section S4. Cuprosolvency testing using fresh copper solids
The cuprosolvency method using fresh copper solids was modified to overcome the orthophosphate demand of the aging solids. The modified method utilized a 1L batch of test water (pH 7.5, 250 mg/L as $CaCO_3$ alkalinity, 0.2 mg/L NOM, and 200 mg/L sulfate) and either one initial 4.3 mg/L as P dose of orthophosphate, or the same total orthophosphate added throughout the test to maintain a residual of about 1.5 mg/L as P. These dosing strategies yielded variable cuprosolvency results in comparison to previous cuprosolvency test methods (**Figure S13**).

When successive dosing was performed, orthophosphate was added to maintain a concentration of about 1.5 mg/L as P. Results demonstrate initial depletion of orthophosphate coupled with reductions in soluble copper (**Figure S13**), as may be expected if cupric phosphate solids were rapidly forming and controlling cuprosolvency.[3, 4] Orthophosphate and copper concentrations remained relatively stable after 2 hours of reaction time, confirming that a steady state condition was achieved much faster than in the tube tests that first require a reservoir of cupric solids to form. The final dissolved copper concentration of 0.53 mg/L (17 hours) was just over half that in the copper tube-based test (0.96 mg/L after 35 weeks) with similar conditions and residual orthophosphate concentration. The additional orthophosphate and potential faster kinetics in this test may have facilitated more complete formation of a cupric phosphate scale than in previous tube or fresh solids tests.

In contrast, when the same total orthophosphate (4.3 mg/L as P) was added as one initial dose, residual orthophosphate quickly fell below the 1.5 mg/L as P target, yielding almost three times higher copper (1.41 mg/L dissolved copper, 5 hours) than when the target residual was maintained at a constant level during the experiment. Orthophosphate could have been limiting in both tests, with less than half the orthophosphate added than is stoichiometrically needed to form cupric phosphate. The higher residual orthophosphate and lower cuprosolvency in the successive dosing test could result from formation of larger particles, reducing the available surface area for reaction with orthophosphate. Overall, these results demonstrate that the timing and dose of orthophosphate addition can affect particle formation via orthophosphate uptake and cuprosolvency. Further, they highlight the need for developing a standardized particle cuprosolvency test method that accounts for factors like solution mixing and maintaining a target residual.

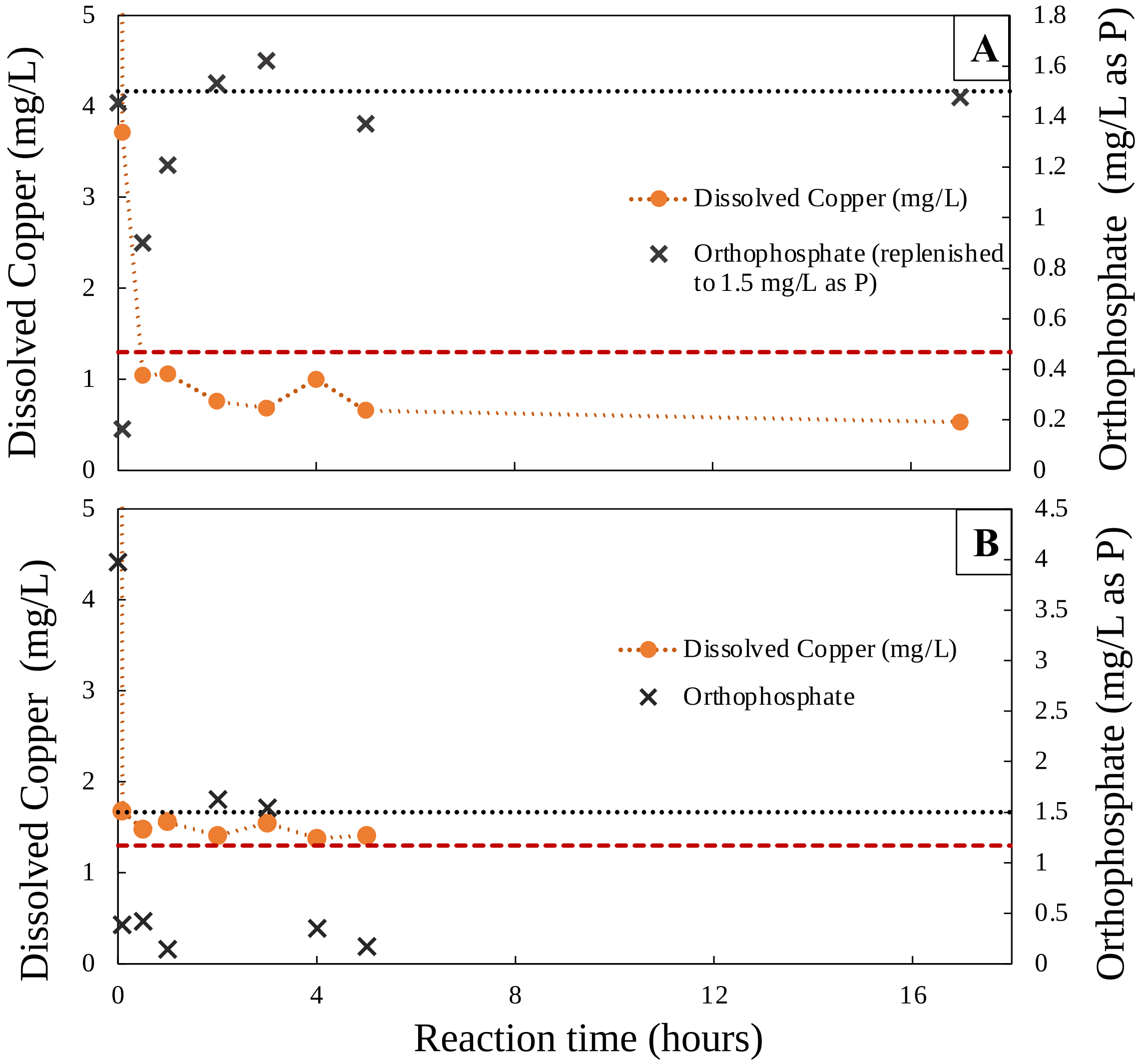

Figure S13. Dissolved copper release from copper particles treated with 4.3 mg/L as P orthophosphate corrosion control A. Added to replenish orthophosphate concentration to 1.5 mg/L as P and B. Added initially all at once. Initial copper concentrations are 0.5mM or 31.8 mg/L. Red dotted lines denote the 1.3 mg/L action level. Black dotted lines denote the target residual orthophosphate concentration. All orthophosphate (OP) concentrations represent the residual measured at given sampling times and are in mg/L as P.

References

(1) Lytle, D. A.; Schock, M. R.; Leo, J.; Barnes, B. A model for estimating the impact of orthophosphate on copper in water. *Journal- American Water Works Association* **2018**, *110* (10), E1-E15.

(2) Kriss, R.; Edwards, M. Challenges Controlling Cuprosolvency in Drinking Water Using "Minimum pH" Criteria. *ACS ES&T Water* **2023**, *3* (11), 3554-3562.

(3) Schock, M. R.; Lytle, D. A.; Clement, J. A. *Effect of pH, DIC, orthophosphate and sulfate on drinking water cuprosolvency*; United States Environmental Protection Agency, National Risk Management Research Laboratory, Cincinnati, OH, 1995.

(4) Edwards, M.; Powers, K.; Hidmi, L.; Schock, M. The role of pipe ageing in copper corrosion by-product release. *Water Science and Technology: Water Supply* **2001**, *1* (3), 25-32.
